# The Alterations of Cortical Volume, Thickness, Surface, and Density in the Intermediate Sporadic Parkinson's Disease from the Han Population of Mainland China

**DOI:** 10.3389/fnagi.2016.00185

**Published:** 2016-08-03

**Authors:** Xia Deng, Meihong Zhou, Chunyan Tang, Jie Zhang, Lei Zhu, Zunchun Xie, Honghan Gong, Xiangzuo Xiao, Renshi Xu

**Affiliations:** ^1^Department of Neurology, First Affiliated Hospital of Nanchang UniversityNanchang, China; ^2^Department of Biochemistry and Molecular Biology, College of Basic Medical Science, Nanchang UniversityNanchang, China; ^3^Department of Radiology, First Affiliated Hospital of Nanchang UniversityNanchang, China

**Keywords:** sporadic Parkinson's disease, magnetic resonance image, cortical morphometry, Han population, Mainland China

## Abstract

Many symptoms of sporadic Parkinson's disease (sPD) can't be completely explained by the lesion of simple typical extrapyramidal circuit between striatum and substantia nigra. Therefore, we investigated the alteration of cortical volume, thickness, surface, and density in the intermediate sPD from the Han population of Mainland China in order to find the new pathological brain regions associated with the complex clinical manifestations of sPD. The cortical volume, thickness, surface and density were examined using the voxel-based cortical morphometry and corticometry on magnetic resonance image (MRI) in 67 intermediate sPD and 35 controls, the multiple adjusted comparisons analysis of all MRI data were employed to assess the relationships between the cortical morphometric alteration in the specific brain regions and sPD. Results showed that a significantly shrunk volume, thinned thickness and enlarged or reduced surface of cortex in some specific brain regions were closely associated with sPD, but all cortical densities were not different. The majority of morphometric alteration of hemisphere cortex was symmetric, but that in the left hemisphere was more significant. The cortical morphometric alterations in the frontal, temporal, parietal, occipital and limbic lobe, cerebellum, caudate, and thalamus were closely related to the clinical neural dysfunction (Clinical manifestations) of sPD. Our data indicated that the deficits of extensive brain regions involved in the development of sPD, resulted in a series of correspondent complex clinical manifestations in the disease.

## Introduction

Sporadic Parkinson's disease (sPD) is the commonest neurodegenerative movement disorder. The destruction of dopaminergic neurons in the pars compacta of substantia nigra (SNpc) in midbrain is a pathologic hallmark of sPD (Fearnley and Lees, 1991), which contributes to the altered activity in the typical extrapyramidal circuit between striatum and SNpc, generates an insufficiency of dopamine neurotransmitter to induce a series of clinical motor manifestations including tremor, rigidity, bradykinesia and the postural instability (Jellinger, 2002; Lehéricy et al., 2004a,b). Besides the typical motor symptoms, there are a lot of non-motor symptoms which appear in any stages of the disease including the early, intermediate (progressive), and late courses, such as hyposmia (Olfactory deficits), the sebaceous gland hyperfunction, the orthostatic hypotension, the excessive sweat secretion, coprostasis, anxiety, depression, the autonomic neural disorders, the sensory dysfunctions, the sleep disturbances, the behavioral problems, the cognitive impairments and decline (Stacy, 2011) and so on, the majority of these no motor symptoms can't been completely explained by the lesion of SNpc and striatum circuit, among them, the partial symptoms have been determined to be associated with the impairment of some cortex and/or white fascicule function of brain regions outside SNpc and striatum circuit in the progression of sPD through the technology of functional magnetic resonance imaging (fMRI) (Monchi et al., 2001, 2004; Huang et al., 2007). However, the sPD-related brain regions of localized cortical degeneration remain completely unclear and need be further comprehensively investigated.

At present, the voxel based morphometry (VBM) is a technique based on the delineation of cortex and normalization, can assess the cortical atrophy including the cortical volume, thickness, surface and density (Ashburner and Friston, 2000). It has been applied by many researchers to investigate various regions of cortical atrophy in sPD. In several studies of non-demented sPD population compared to the healthy controls, revealed the cortical atrophy in the left anterior cingulated (Summerfield et al., 2005), the left rectus and the parahippocampal gyrus and the right inferior frontal gyrus (Nagano-Saito et al., 2005) and the right frontal lobe (BA 45, 47, 10; Burton et al., 2004), but couldn't completely explain these clinical variabilities of sPD and showed the lack of a consistent portrait of damage on the cerebral cortex with the complex clinical manifestations (Davatzikos, 2004). In addition, a structural analysis technique using the MRI anatomical scans known as corticometry has also recently been used in investigating the local cortical thickness and surface. The local cortical thickness reflects the local cortical volume of VBM notion. The analysis of local cortical surface assesses the local folding and gives access to the cortical geometrical properties. The analysis of combined VBM and corticometry techniques allows for enhancing reliability and sensitivity in investigating the alteration of cortical volume, thickness, surface and density (Jubault et al., 2011). The alteration of cortical volume, thickness and surface can indirectly assess the cortical atrophy of the distinct regions. Therefore, in this study, we studied the alteration of cortical volume, thickness, surface and density in the intermediate sPD from the Han population of Mainland China at present (HPCM) using VBM and corticometry techniques.

We hypothesized that the construct of local cortical regions would exhibit alterations due to sPD, which was involved in the generation of motor and/or no motor symptoms. Furthermore, MRI studies have revealed some alteration of local cortical regions in sPD (Burton et al., 2004; Nagano-Saito et al., 2005; Summerfield et al., 2005). Based on our hypothesis and the previous studied evidences, we expected to find some distinct damaged brain regions being consistent with the clinical manifestations and the local brain lesion closely related to generate the special clinical manifestations in the intermediate sPD patients.

Braak et al. (2003) have proposed a 6-stages model of sPD neuropathology progression, in the process, the cortical pathology would begin in the temporal mesocortex regions (stage 4), continue in the prefrontal cortex (stage 5), and finally in the primary sensory and the premotor areas (stage 6). Therefore, we expected to reveal the alteration of cortical volume, thickness, surface and density in some local functional regions of brain related to the intermediate stage of sPD through the combined VBM and corticometry techniques. This study aimed to address the alteration of cortical volume, thickness, surface, and density in the progressive (Intermediate) stage of sPD from HPCM, to find the relations between the local structure alteration of brain functional regions and the clinical manifestations of sPD, to identify a stable pattern of brain local functional alteration being consistent with the clinical manifestations in sPD, to provide some objective evidences such as the brain morphometric alterations of MRI for the diagnosis of sPD, and to explore some new potential pathological lesion related with sPD (Supplemental Figure 1).

## Materials and methods

### Participants

This study included 100 intermediate sPD (Progressive or Advanced sPD) and 40 control subjects recruited from the First Affiliated Hospital of Nanchang University in the HPCM from July 2011 to December 2014. Among them, 67 patients and 35 controls were underwent a finial investigation, 33 patients and 5 controls were excluded from further analysis due to the technical artifacts/factors. All sPD participants met the UK brain bank criteria for the diagnosis of sPD (Hughes et al., 1992). In order to have a more precise measure of disease severity, we administered the motor subset of the modified Hoehn and Yahr and the UPDRS-III scale in the sPD group. The sPD patients who have the 2.5–3 stage of modified Hoehn and Yahr and the 31–45 score of UPDRS-III were recruited as the intermediate sPD. Table 1 gave the clinical and demographic details of sPD and the control groups. All scale and diagnosis were administered by three experienced neurologists. All procedures were performed under protocols approved by the First Affiliated Hospital of Nanchang University in Nanchang, Jiangxi, China. In all cases, a written informed consent for research was obtained from the patient or the legal guardian, and the used material had an appropriate ethical approval for using in this project.

**Table 1:** **Demographics and disease-related characteristics in sPD and matched controls**.

**Demographics/Characteristics**	**sPD** ***N* = 67**	**Controls** ***N* = 35**	**Honmo-geneity of variance test *F*-value**	***t*-value**	**Welch** ***t*′-value**	***P*-value**
**DEMOGRAPHICS**						
Age, years	65.31 ± 5.67	67.3 ± 5.09	1.24	−1.74		0.08493551
Gender, M/F	37/30 (M/F)	24/11 (M/F)		χ2 = 1.19		0.2753
Education, years	11.7 ± 5.50	12.74 ± 5.94	1.17	−0.88		0.38096922
Disease duration, years	8.1 ± 1.17	N/A				
**DISEASE SEVERITY**						
Hoehn and Yahr scale	2.7 ± 0.3	N/A				
UPDRS Part I- mood and cognition	8.67 ± 1.37	N/A				
UPDRS Part II-activities of daily living	28.82 ± 1.59	N/A				
UPDRS Part III- motor examination	34.11 ± 2.0	N/A				
UPDRS Part IV- medication complications	11.57 ± 1.38	N/A				
Total UPDRS score (sum of Parts I-IV)	83.17 ± 6.34	N/A				
Symptom-dominant side (right/left/double)	16/44/7	N/A				
Tremor subscoree off (e)	3.37 ± 0.74	N/A				
Tremor subscoref on (f)	2.26 ± 0.64	N/A				
Webster	15.67 ± 2.98	N/A				
MMSE	15.00 ± 2.47	29.5 ± 0.8	9.53		−43.86	4.62719E-62
HDS-R	16.80 ± 3.82	29.6 ± 2.35	2.64		−20.88	3.07271E-38
DF	5.8 ± 1.51	9.2 ± 2.1	1.93	76.49	−8.5	1.55129E-11
DB	3.8 ± 1.44	6.5 ± 2.1	2.13	−28.72	−6.815	9.76169E-09
SVFT	14.25 ± 4.40	17.3 ± 2.8	2.47		−4.26	4.66304E-05
SDS	66.20 ± 7.16	28.2 ± 3.4	4.43		36.33	8.48488E-60
HAMD 17	34.20 ± 5. 63	2.6 ± 2.4	5.5		39.67	1.38241E-62
HAMD 24	46.6 ± 2.80	3.0 ± 2.6	1.16		1.98	1.49963E-90
CDT	2.25 ± 0.26	3.9 ± 0.3	1.33			4.18693E-50
CDR	0.49 ± 0.34	0.38 ± 0.33	1.33			0.05160411
LEDD	568.97 ± 308.63	N/A				
PDSI	12.2 ± 3.69	N/A				
**CARDINAL MOTOR SYMPTOMS**						
Tremor,	67	N/A				
Rigidity	67	N/A				
Bradykinesia	67	N/A				
Postural instability	67	N/A				

*Abbreviation: MMSE, mini mental state examination; HDS-R, Hasegawa dementia scale revised; DF, the forward digit span task; DB, the backward digit span task; SVFT, semantic verbal fluency test; SDS, self-rating depression scale; HAMD, Hamilton depression scale; CDT, clock drawing task; CDR, clinical dementia rating; LEDD, Levodopa (l-dopa) equivalent daily doses; PDSI, the PD screening instrument scores. N/A, not applicable*.

*Comment, HAMD17 represents the sum of the previous 17 Hamilton anxiety scale item; HAMD24 represents the sum of the previous 24 Hamilton anxiety scale item; Tremor subscoree off (e) represents the sum of the following unified Parkinson's Disease rating scale (III) item: 20. Tremor subscoref on (f) represents the sum of the following unified Parkinson's Disease rating scale (III) item: 21. LEDD = [levodopa (× 1.2 if catechol-O-methyltransferase (COMT) inhibitor) (× 1.2 if 10 mg of selegiline or × 1.1 if 5 mg of selegiline)] + [pramipexole × 400] + [Ropinirole × 40] + [Cabergoline × 160]+ [pergolide × 200] + [bromocriptine × 10] + [lisuride × 160], all doses are in mg*.

### MRI acquisition

The data of high-resolution structural MRI were acquired from a 3.0 tesla Siemens Tim Trio MRI scanner, Participants were scanned using a three-dimensional T1-weighted MPRAGE sequence at the Department of Radiology, the First Affiliated Hospital of Nanchang University. The scanning parameters were as follows: TR/TE/TI: 1900/2.26/900 ms, the flip angle: 9°, the slice thickness 1 mm, 176 slices, the field of view 256 × 256 mm^2^, the acquisition matrix 256 × 256, the voxel size: 1 × 1 × 1 mm^3^, 8-channels coil. Structural MRI series included T1-weighted 3D fast, spoiled gradient recalled echo images and other sequences such as T2-weighted and FLAIR images to visualize focal lesions of cortical or white matter that might be exclusionary.

### Image processing

All image processing were performed by the State Key Laboratory of Cognitive Neuroscience and Learning and IDG/McGovern Institute for Brain Researching, Beijing Normal University, China. Briefly, the CIVET pipeline was used to measure the cortical volume, thickness, surface and density on VBM and corticometry as previously described (Feldmann et al., 2008a; Jubault et al., 2011; Gong et al., 2012; Xia et al., 2013; Chen et al., 2015). The native T1-weighted MRI were first linearly aligned into the stereotaxic space and corrected for non-uniformity artifacts using the N3 algorithm (Sled and Pike, 1998). The resultant brain images were then automatically segmented into cortex, white matter, CSF, and background by using a partial volume (PV) classification algorithm, in which a trimmed minimum covariance determinant method was applied for estimating the parameters of the PV effect model; the parameter β controlling the relative strength of the Markov random field was set to 0.1 (Tohka et al., 2004). Next, the inner and outer cortical surfaces were automatically extracted for each hemisphere using the CLASP algorithm (Kim et al., 2005). The individual surfaces were further aligned with a surface template to allow comparisons across subjects at corresponding vertices. The cortical surfaces of the inner and outer cortex which consisted of 40,962 vertices were extracted automatically using the Constrained Laplacian-based Automated Segmentation with Proximities (CLASP) algorithm (MacDonald et al., 2000). The cortical surfaces were inversely transformed to native space. Cortical thickness was measured between the two surfaces at 40,962 vertices per hemisphere using the linked distance in the native space (Lerch and Evans, 2005). Cortical thickness was defined using the link method, which measures the Euclidean distance between the linked vertices of the inner and outer surfaces (MacDonald et al., 2000; Im et al., 2006). The middle cortical surface, defined at the geometric center between the inner and outer cortical surfaces, was used to calculate the cortical surface in the native space (Lyttelton et al., 2009). The thickness/surface map was further blurred with a 30 mm surface-based diffusion smoothing kernel (Chung et al., 2003). The vertex-wise sphere-to-sphere warping nonlinear surface registration was performed to unbiased iterative surface template (Lerch and Evans, 2005). Using the surface registration, the thickness information on native surfaces was transformed to a template after diffusion smoothing with 20-mm full-width half maximum to increase the signal-to-noise ratio and improve the detection ability of population changes (Kabani et al., 2001; Im et al., 2006; Hong et al., 2014). All cortical image processing was conducted by investigators blinded to the patient demographics, disease and controls.

### Statistical analysis

We used one-way analyses of variance (ANOVA) with the *post-hoc* Bonferroni correction to examine for differences in age, education, the PD duration, MMSE, HDS-R, DF, DB, SVFT, SDS, HAMD17, HAMD 24, CDT, CDR, the mean cortical volume, thickness, surface and density values in the regions of interest between sPD and control groups. A chi-squared test was used to assess for differences in sex distribution between the groups.

In addition, all cortical analysis was finished by the State Key Laboratory of Cognitive Neuroscience and Learning and IDG/McGovern Institute for Brain Research, Beijing Normal University, China. The statistical analysis of cortical volume, thickness, surface and density was performed in the vertex-wise level by an analysis of covariance (ANCOVA) using the brain size, the cortical thickness or the cortical surface as covariates for comparisons among the groups. A correction for the comparison of the cortical volume between the sPD subjects and the controls was conducted at a corrected probability value of *p* < 0.01 and a lowered discriminative threshold of *p* < 0.001. To correct for the multiple vertex-wise comparisons, a random field theory (RFT)-based method was applied at the cluster level (Taylor and Adler, 2003), and the cortical clusters surviving a Family wise error (FEW)-corrected *p* < 0.05 were considered as significant. All these statistical procedures were implemented using SurfStat toolbox (http://www.math.mcgill.ca/keith/surfstat/). All names of anatomical regions were cited from Anatomical Automatic Labeling (AAL) of Montreal Neurological Institute (MNI). The significance difference and the correlation maps were created as applicable. The maps were corrected for multiple comparisons with permutation analysis at a threshold *p* < 0.05, *p* < 0.01, or *p* < 0.001. An uncorrected comparison of cortical volume, thickness and surface between sPD and control also was performed by Student's *t*-test with the significant of *p* ≤ 0.05. The value of cortical volume, thickness, surface, and density were expressed as mean ± s.d.

## Results

### Clinical characteristics

The details of demographics and disease-related characteristics in sPD and the matched controls were seen in Table 1. the intermediate sPD indicated that the sPD patient must have all 4 major clinical signs including tremor, rigidity, bradykinesia and the postural instability, and the dopaminergic reflection was the best, the Hoen and Yahr scale was 2.7 ± 0.3, Total UPDRS score (Sum of Parts I–IV) was 83.17 ± 6.34, UPDRS Part III score (Motor examination) was 34.11 ± 2.0 (Table 1).

### Abnormal brain regions of cortical volume in sPD patients vs. control

**Figure 1:**
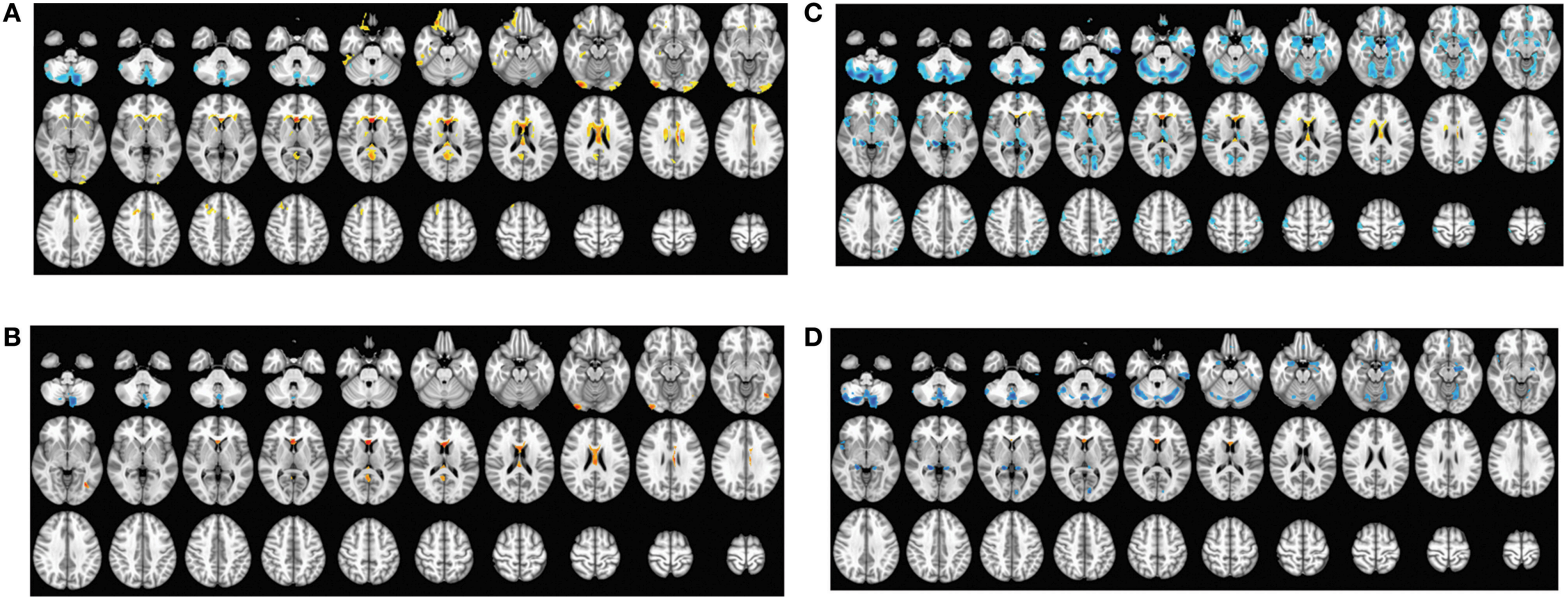
**The comparison of cortical volume between sPD and the control using the regression of different covariates. (A)** The significantly altered brain regions in the analysis of the regression of no covariates with a threshold *p* < 0.01. **(B)** The significantly altered brain regions in the analysis of the regression of no covariates with a threshold *p* < 0.001. **(C)** The significantly altered brain regions in the analysis of the regression of brain size covariates with a threshold *p* < 0.01. **(D)** The significantly altered brain regions in the analysis of the regression of brain size covariates with a threshold *p* < 0.001.

The cortical volumes were significantly diminished in the patients with sPD compared with the normal controls through analyzing by the regression of no covariates and the regression of brain size covariates (Table 2, Figure 1). The brain regions showed the cortical volume shrinkage through analyzing by the regression of no covariates included Frontal lobe (Frontal-Sup-Orb-L (2 brain regions), Frontal-Mid-Orb-L (1), Frontal-Inf-Orb-L (2), Frontal-Inf-Oper-L(1), Frontal-Sup-L(1), Frontal-Mid-L(1) and Frontal-Sup-Medial-L(1), Rectus-L(2), Precentral-L(1)); Temporal lobe (Temporal-Pole-Sup-L(1), Olfactory-L(1), Olfactory-R(1), Calcarine-R(2)); Parietal lobe (Precuneus-L(1), Precuneus-R(1)); Occipital lobe (Occipital-Mid-R(1), Occipital-Inf-R(1), Lingual-L(1), Lingual-R(2)); Limbic lobe (Hippocampus-L(1), Hippocampus-R(1), Insula-L(1), Cingulum-Ant-L(1), Cingulum-Ant-R(1), Cingulum-Mid-L(1), Cingulum-Mid-R(1), Cingulum-Post-L(1), Cingulum-Post-R(1), Calcarine-L(1)); Cerebellum-Crus1-R (1), Vermis-4-5(1), Caudate-L(1), Caudate-R (1), Thalamus-L(1), Thalamus-R(1) gyrus. Among them, the most significant brain regions were Frontal-Sup-Orb-L, Frontal-Mid-Orb-L, Frontal-Inf-Orb-L, Frontal-Sup-L, Frontal-Mid-L, Frontal-Sup-Medial-L, Rectus-L, Precuneus-L, Precuneus-R, Lingual-L, Hippocampus-L Insula-L, Cingulum-Ant-L, Cingulum-Ant-R, Cingulum-Mid-L, Cingulum-Mid-R, Cingulum-Post-L, Cingulum-Post-R, and Olfactory-L gyrus (Figures 1A,B).

**Table 2:** **Differences of cortical volume within the significant regions between sPD and control**.

	**Coordinates**			**Voxel**	**Cortical volume**		***P*-value**
	***X***	***Y***	***Z***		**sPD**	**Control**	
**REGIONS OF INTEREST (REGRESSION OF NO COVARIATES) (sPD<CONTROL)**							
**Cluster 2**							
Frontal-Sup-Orb-L^*^	−70.5	161	49.5	366	0.3294 ± 0.055	0.4423 ± 0.0407	0.000489^*^
Frontal-Mid-Orb-L^*^	−69	161	49.5	76	0.3607 ± 0.0851	0.5198 ± 0.0393	<.0001^*^
Frontal-Inf-Orb-L^*^	−58.5	153	51	437	0.3707 ± 0.0972	0.517 ± 0.0493	<.0001^*^
Rectus-L^*^	−78	176	55.5	24	0.2408 ± 0.0347	0.3088 ± 0.0326	0.006644^*^
Temporal-Pole-Sup-L	−58.5	153	48	6	0.2026 ± 0.0977	0.3205 ± 0.1844	0.47404
**Cluster 3**							
Calcarine-R	−111	24	67.5	4	0.2049 ± 0.1573	0.3139 ± 0.4633	0.085352
Lingual-R	−114	33	63	199	0.2635 ± 0.1061	0.4089 ± 0.2047	0.499524
Occipital-Mid-R	−117	37.5	72	1	0.2278 ± 0.1037	0.294 ± 0.2994	0.095468
Occipital-Inf-R	−114	31.5	61.5	568	0.2929 ± 0.0969	0.4426 ± 0.156	0.268733
Cerebelum-Crus1-R	−118.5	40.5	54	2	0.3987 ± 0.1237	0.7192 ± 0.3323	0.142919
**Cluster 4**							
Frontal-Sup-Orb-L	−73.5	153	60	3	0.087 ± 0.0046	0.1143 ± 0.0064	0.141939
Frontal-Inf-Oper-L	−48	140	84	50	0.1018 ± 0.006	0.1364 ± 0.0091	0.208735
Frontal-Inf-Orb-L^*^	−67.5	153	63	12	0.1283 ± 0.0307	0.177 ± 0.0107	<.0001^*^
Olfactory-L^*^	−88.5	149	72	42	0.2346 ± 0.032	0.3224 ± 0.0165	<.0001^*^
Olfactory-R	−96	153	72	11	0.1019 ± 0.0137	0.1457 ± 0.0167	0.058702
Rectus-L^*^	−76.5	153	61.5	12	0.1933 ± 0.0148	0.2598 ± 0.0098	0.000106^*^
Insula-L^*^	−61.5	141	85.5	44	0.1381 ± 0.0163	0.201 ± 0.01	0.000423^*^
Cingulum-Ant-L^*^	−81	155	66	125	0.3029 ± 0.0664	0.3986 ± 0.029	<.0001^*^
Cingulum-Ant-R^*^	−100.5	153	88.5	273	0.3013 ± 0.0777	0.4133 ± 0.0541	0.000216^*^
Cingulum-Mid-L^*^	−84	135	105	1	0.3056 ± 0.0698	0.3957 ± 0.0313	<.0001^*^
Cingulum-Mid-R^*^	−100.5	114	105	437	0.2674 ± 0.0484	0.3667 ± 0.0161	<.0001^*^
Cingulum-Post-L^*^	−88.5	81	87	167	0.3658 ± 0.0815	0.4722 ± 0.0309	<.0001^*^
Cingulum-Post-R^*^	−99	88.5	84	75	0.1596 ± 0.0189	0.2408 ± 0.0357	<.0001^*^
Hippocampus-L^*^	−76.5	88.5	84	6	0.0907 ± 0.0053	0.1303 ± 0.018	0.031711^*^
Hippocampus-R^*^	−111	93	81	50	0.2087 ± 0.032	0.2989 ± 0.1087	0.035585^*^
Calcarine-L	−88.5	69	85.5	13	0.3573 ± 0.0665	0.6142 ± 0.2012	0.073437
Calcarine-R	−91.5	69	85.5	31	0.4398 ± 0.1136	0.7075 ± 0.1385	0.058702
Lingual-L^*^	−88.5	72	79.5	1	0.2356 ± 0.076	0.4381 ± 0.4531	0.000265^*^
Lingual-R	−96	72	81	1	0.4024 ± 0.1466	0.6639 ± 0.2093	0.153643
Precuneus-L^*^	−88.5	73.5	85.5	488	0.4761 ± 0.103	0.6696 ± 0.0545	<.0001^*^
Precuneus-R^*^	−94.5	73.5	87	215	0.4028 ± 0.0845	0.5639 ± 0.0325	<.0001^*^
Caudate-L	−70.5	129	96	194	0.2682 ± 0.0515	0.3765 ± 0.1175	0.293891
Caudate-R^*^	−108	152	84	16	0.1417 ± 0.0886	0.2549 ± 0.5114	0.000359^*^
Thalamus-L	−76.5	117	87	55	0.2067 ± 0.0268	0.2721 ± 0.0471	0.374341
Thalamus-R	−109.5	94.5	81	3	0.2501 ± 0.0474	0.3371 ± 0.0955	0.448204
Vermis-4-5	−88.5	75	79.5	10	0.2286 ± 0.1132	0.4142 ± 0.2806	0.180383
**Cluster 5**							
Precentral-L	−58.5	128	129	46	0.4149 ± 0.1841	0.6084 ± 0.2358	0.082983
Frontal-Sup-L^*^	−69	144	127.5	499	0.3294 ± 0.1212	0.4644 ± 0.0352	<.0001^*^
Frontal-Mid-L^*^	−60	129	129	1052	0.366 ± 0.1326	0.5317 ± 0.08	<.0001^*^
Frontal-Sup-Medial-L^*^	−79.5	147	114	9	0.2844 ± 0.0589	0.416 ± 0.1929	0.04479^*^
**REGIONS OF INTEREST (REGRESSION OF BRAIN SIZE COVARIATES) (sPD<CONTROL)**							
**Cluster 1**							
Lingual-R	−109.5	48	61.5	286	0.4938 ± 0.1178	0.7125 ± 0.145	0.061332
Fusiform-L^*^	−48	79.5	46.5	39	0.4648 ± 0.1079	0.7634 ± 0.4646	0.005685^*^
Fusiform-R	−109.5	73.5	60	91	0.5304 ± 0.1322	0.8045 ± 0.2115	0.259457
Temporal-Inf-L^*^	−46.5	81	45	21	0.383 ± 0.0952	0.6262 ± 0.9143	<.0001^*^
Cerebelum-Crus1-L^*^	−48	73.5	43.5	942	0.5015 ± 0.1871	0.66 ± 0.2226	0.049189^*^
Cerebelum-Crus1-R^*^	−112.5	52.5	42	860	0.5727 ± 0.1415	0.7343 ± 0.1118	0.00106^*^
Cerebelum-Crus2-L	−67.5	52.5	27	1533	0.4502 ± 0.0949	0.5724 ± 0.2939	0.063124
Cerebelum-Crus2-R^*^	−105	48	28.5	1172	0.5297 ± 0.1595	0.6355 ± 0.1464	0.005226^*^
Cerebelum-4-5-L^*^	−70.5	73.5	54	45	0.6006 ± 0.1826	0.8753 ± 0.2099	0.037578^*^
Cerebelum-4-5-R	−108	72	60	176	0.5562 ± 0.1822	0.8071 ± 0.3791	0.399803
Cerebelum-6-L^*^	−48	81	45	878	0.6037 ± 0.1847	0.8372 ± 0.1205	<.0001^*^
Cerebelum-6-R^*^	−112.5	55.5	43.5	1243	0.5986 ± 0.1857	0.8241 ± 0.1106	<.0001^*^
Cerebelum-7b-L	−69	54	27	881	0.5206 ± 0.1758	0.6058 ± 0.3313	0.460967
Cerebelum-7b-R	−105	49.5	28.5	425	0.5511 ± 0.2886	0.6336 ± 0.3899	0.111283
Cerebelum-8-L	−61.5	63	24	1162	0.5341 ± 0.2167	0.5996 ± 0.446	0.411603
Cerebelum-8-R	−106.5	54	28.5	1268	0.5126 ± 0.2533	0.5898 ± 0.5734	0.303459
Cerebelum-9-L^*^	−90	70.5	30	46	0.5578 ± 0.4862	0.6425 ± 0.4494	0.005754^*^
Cerebelum-9-R^*^	−94.5	64.5	30	81	0.515 ± 0.5234	0.6084 ± 0.5301	0.013566^*^
Vermis-4-5^*^	−97.5	64.5	61.5	19	0.4545 ± 0.1311	0.6142 ± 0.572	0.005079^*^
Vermis-6^*^	−94.5	63	61.5	7	0.3736 ± 0.1169	0.5097 ± 0.6321	0.000715^*^
Vermis-7^*^	−94.5	57	40.5	221	0.4535 ± 0.069	0.5905 ± 0.3492	0.001354^*^
Vermis-8^*^	−94.5	61.5	36	441	0.4925 ± 0.1821	0.5649 ± 0.6346	0.029364^*^
Vermis-9	−94.5	64.5	33	215	0.5245 ± 0.3254	0.5942 ± 0.4403	0.115954
Vermis-10	−90	78	37.5	4	0.3764 ± 0.2069	0.4385 ± 0.3721	0.410331
**Cluster 2**							
Hippocampus-R	−108	114	57	618	0.5809 ± 0.1077	0.7122 ± 0.2914	0.137848
ParaHippocampal-R	−105	119	55.5	362	0.5246 ± 0.1234	0.6355 ± 0.2655	0.365677
Amygdala-R	−108	128	55.5	342	0.5295 ± 0.1425	0.6615 ± 0.3432	0.233289
Temporal-Pole-Sup-R^*^	−120	131	49.5	13	0.4435 ± 0.1551	0.5599 ± 0.6814	0.004712^*^
**Cluster 3**							
Frontal-Mid-Orb-L^*^	−91.5	177	60	198	0.3522 ± 0.0844	0.475 ± 0.2808	0.039927^*^
Frontal-Mid-Orb-R^*^	−93	176	58.5	287	0.4616 ± 0.143	0.579 ± 0.1361	0.00767^*^
Rectus-L	−91.5	161	55.5	200	0.5006 ± 0.205	0.6487 ± 0.2984	0.172704
Rectus-R	−93	161	54	234	0.4742 ± 0.1572	0.5799 ± 0.1889	0.05375
**Cluster 4**							
Olfactory-L	−70.5	131	55.5	5	0.279 ± 0.1066	0.3762 ± 0.3229	0.073437
Hippocampus-L	−73.5	113	57	655	0.529 ± 0.0812	0.6632 ± 0.1946	0.241275
ParaHippocampal-L	−73.5	110	52.5	185	0.4549 ± 0.1047	0.5807 ± 0.164	0.241616
Amygdala-L^*^	−70.5	128	54	378	0.6131 ± 0. 1509	0.7717 ± 0.1685	0.031329^*^
Temporal-Pole-Sup-L	−70.5	131	52.5	64	0.4262 ± 0.1209	0.5468 ± 0.3311	0.128192
Temporal-Inf-L^*^	−52.5	99	60	1	0.2325 ± 0.0376	0.2904 ± 0.215	0.000397^*^
**Cluster 5**							
Cingulum-Ant-R^*^	−100.5	153	88.5	12	0.1319 ± 0.0235	0.1838 ± 0.0242	0.015624^*^
Cingulum-Mid-R^*^	−99	134	103.5	3	0.1535 ± 0.0168	0.208 ± 0.0127	0.000593^*^
Caudate-L	−72	131	97.5	11	0.1932 ± 0.0531	0.2983 ± 0.1432	0.137848
Caudate-R^*^	−108	152	84	5	0.1623 ± 0.1139	0.2982 ± 0.762	<.0001^*^
**Cluster 6**							
Precentral-L	−45	114	130.5	210	0.235 ± 0.105	0.2784 ± 0.3165	0.076257
Postcentral-L	−42	102	135	747	0.2604 ± 0.1349	0.2889 ± 0.2929	0.35474

*X, Y, and Z are in MNI coordinates. For each cluster, we report the highest peak value. Cortical volume is expressed in mm^3^*.

* *Indicates a significance of p ≤ 0.05 uncorrected*.

The brain regions showed the cortical volume shrinkage through analyzing by the regression of brain size covariates included Lingual-R(1), Fusiform-L(1), Fusiform-R(1), Temporal-Inf-L(2), Cerebellum-Crus1-L(1), Cerebellum-Crus1-R(1), Cerebellum-Crus2-L(1), Cerebellum-Crus2-R(1), Cerebellum-4-5-L(1), Cerebellum-4-5-R(1), Cerebellum-6-L(1), Cerebellum-6-R(1), Cerebellum-7b-L(1), Cerebellum-7b-R(1), Cerebellum-8-L(1), Cerebellum-8-R(1), Cerebellum-9-L(1), Cerebellum-9-R(1), Vermis-4-5(1), Vermis-6(1), Vermis-7(1), Vermis-8(1), Vermis-9(1), Vermis-10(1), Hippocampus-R(1), ParaHippocampal-R(1), Amygdala-R(1), Temporal-Pole-Sup-R(1), Frontal-Mid-Orb-L (1), Frontal-Mid-Orb-R(1), Rectus-L(1), Rectus-R(1), Olfactory-L(1), Hippocampus-L(1), ParaHippocampal-L(1), Amygdala-L(1), Temporal-Pole-Sup-L(1), Cingulum-Ant-R(1), Cingulum-Mid-R(1), Caudate-L(1), Caudate-R(1), Precentral-L(1) and Postcentral-L(1) gyrus. Among them, the most significant brain regions were Fusiform-L, Temporal-Inf-L, Cerebellum-Crus1,2-R, Cerebellum-4-5-L, Cerebellum-6-L, Cerebellum-6-R, Cerebellum-9-L, Cerebellum-9-R, Vermis-6, Vermis-7, Vermis-8, Temporal-Pole-Sup-R, and Frontal-Mid-Orb-R gyrus (Figures 1C,D).

The brain regions that the cortical volume exhibited the significant difference between sPD and the controls in the analysis of both the regression of no covariates and the regression of brain size covariates consisted of Frontal-Mid-Orb-L, Rectus-L, Precentral-L, Temporal-Pole-Sup-L, Lingual-R, Hippocampus-L, Hippocampus-R, Cingulum-Ant-R, Cingulum-Mid-R, Olfactory-L, Cerebellum-Crus1-R, Vermis-4-5, Caudate-L and Caudate-R gyrus. The majority of lost hemisphere volume was symmetric, the bilateral symmetrical brain regions consisted of Precuneus, Hippocampus, Cingulum-Ant, Cingulum-Mid, Cingulum-Post, Lingual, Olfactory, Caudate, Thalamus, Fusiform, Cerebellum-Crus1, Cerebellum-Crus2, Cerebellum-4-5, Cerebellum-6, Cerebellum-7b, Cerebellum-8, Cerebellum-9, ParaHippocampal, Amygdala, Temporal-Pole-Sup, Frontal-Mid-Orb and Rectus gyrus, but the cortical volumes in the left hemisphere were significantly diminished (44 of left brain regions vs. 33 of right brain regions). No brain regions with the increased cortical volume were found (Table 2, Figure 1). Among them, the brain regions were compared by the uncorrected average value of cortical volume in the significant different brain regions between sPD and controls, which showed that the significant decreased regions were Frontal-Sup-L, Frontal-Mid-L, Frontal-Sup-Orb-L, Frontal-Mid-Orb-L, Frontal-Mid-Orb-R, Frontal-Inf-Orb-L, Frontal-Sup-Medial-L, Rectus-L, Rectus-R, Temporal-Inf-L, Temporal-Pole-Sup-R, Olfactory-L, Insula-L, Cingulum-Ant-L, Cingulum-Ant-R, Cingulum-Mid-L, Cingulum-Mid-R, Cingulum-Post-L, Cingulum-Post-R, Amygdala-L, Hippocampus-L, Hippocampus-R, Lingual-L, Precuneus-L, Precuneus-R, Fusiform-L, Cerebelum-Crus1-L, Cerebelum-Crus1-R, Cerebelum-Crus2-R, Cerebelum-4-5-L, Cerebelum-6-L, Cerebelum-6-R, Cerebelum-9-L, Cerebelum-9-R, Vermis-4-5, Vermis-6, Vermis-7, Vermis-8, and Caudate-R (Table 2).

### Abnormal brain regions of cortical thickness in sPD patients vs. control

**Figure 2:**
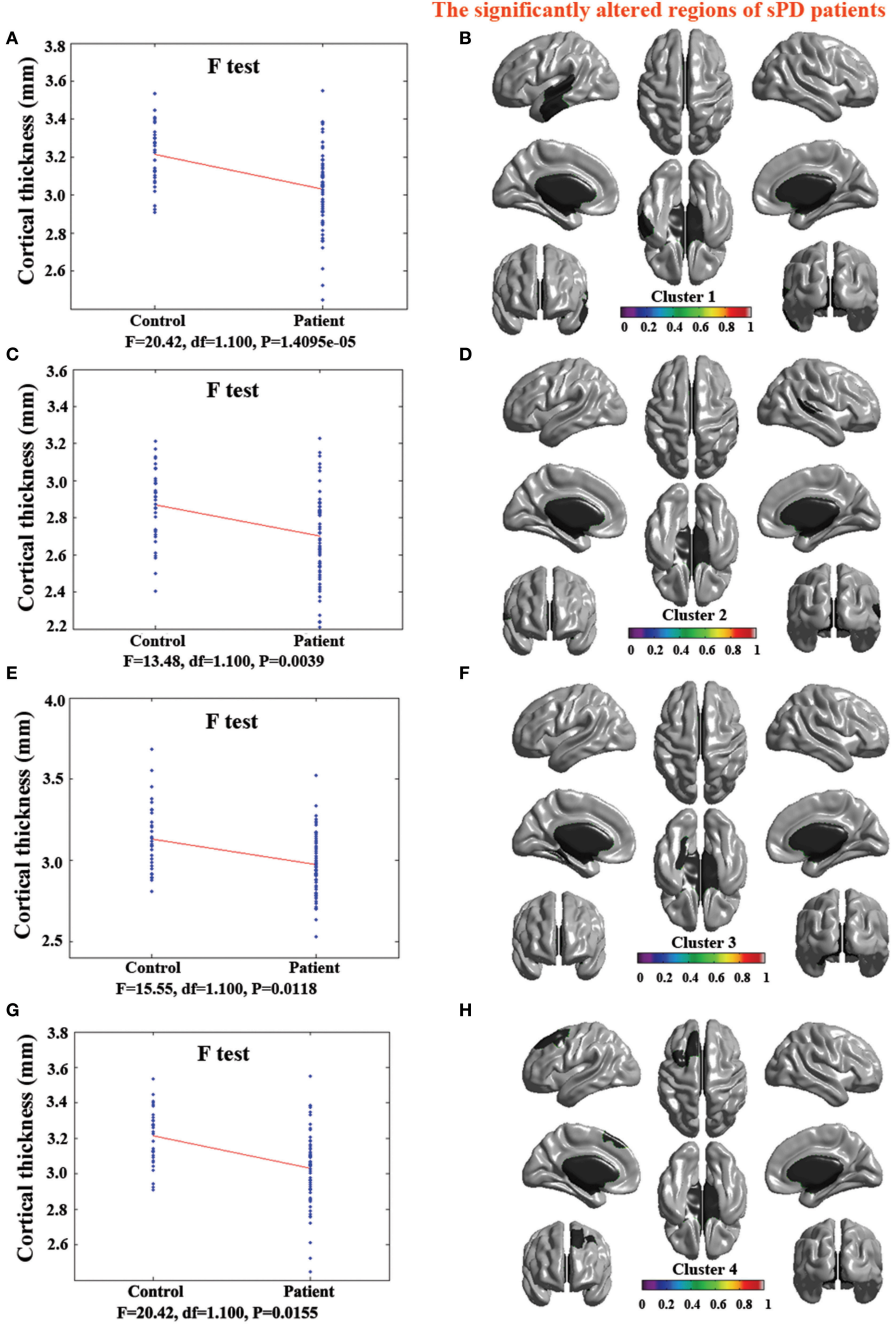
**The comparison of cortical thickness between sPD and the control using the regression of no covariates**. **(A,C,E,G)** The *F*-test of the average cortical thickness in the cluster 1–4 between sPD and the control. **(B,D,F,H)** The significantly altered brain regions in the cluster 1–4 of the sPD brain. All cortical thickness of cluster 1–4 were significantly thinned in sPD compared with the control.

**Figure 3:**
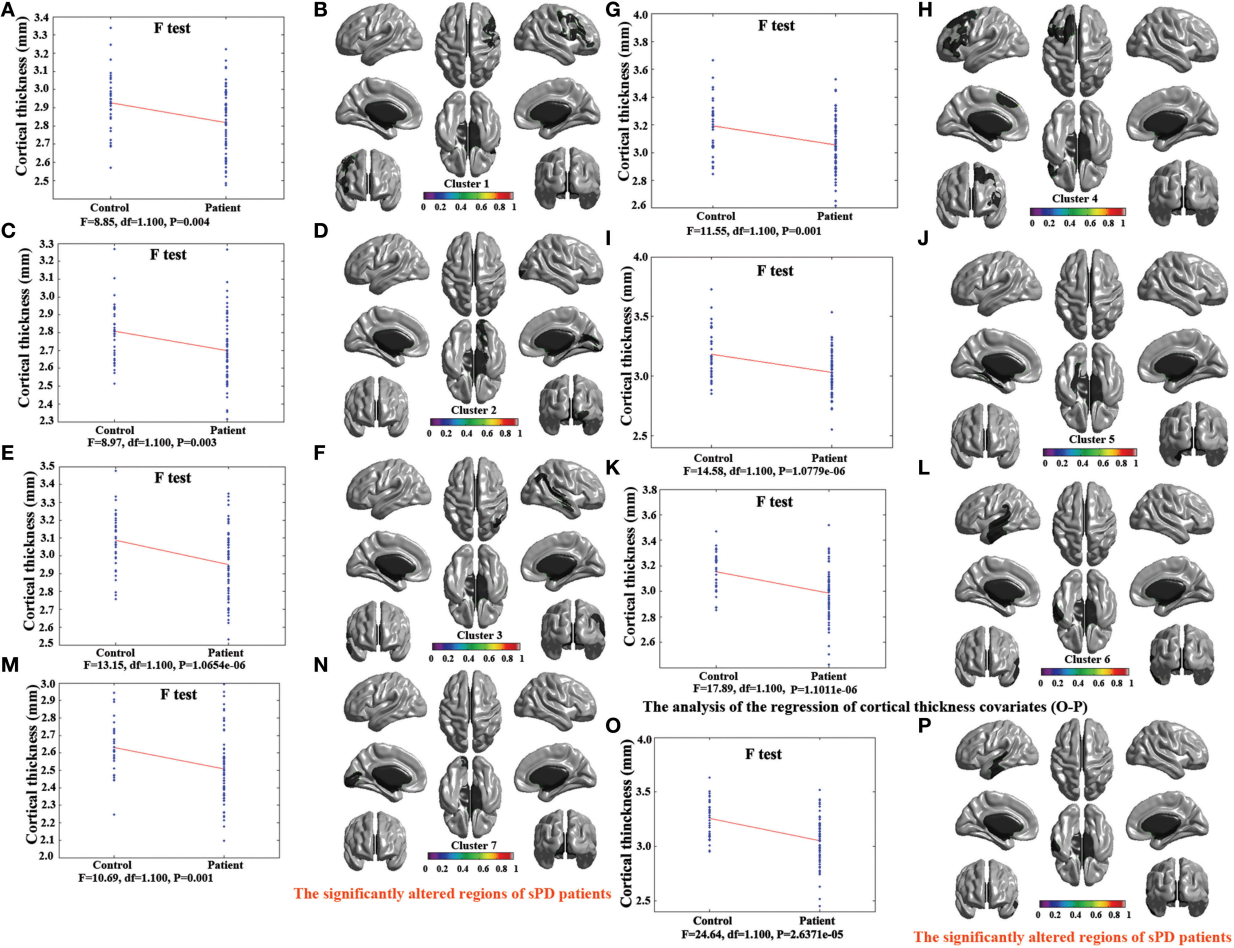
**The comparison of cortical thickness between sPD and the control using the regression of different covariates. (A,C,E,G,I,K,M)** The *F*-test of the average cortical thickness in the cluster 1–7 between sPD and the control in the analysis of the regression of brain size covariates. **(B,D,F,H,J,L,N)** The significantly altered brain regions in the cluster 1–7 of the sPD brain in the analysis of the regression of brain size covariates. **(O)** The *F*-test of the average cortical thickness in the cluster 1 between sPD and the control in the analysis of the regression of cortical thickness covariates. **(P)** The significantly altered brain regions in the cluster 1 of sPD brain in the analysis of the regression of cortical thickness covariates. The cortical thickness of all brain regions were significantly thinned in sPD compared with the control in the analysis using the regression of different covariates.

**Figure 4:**
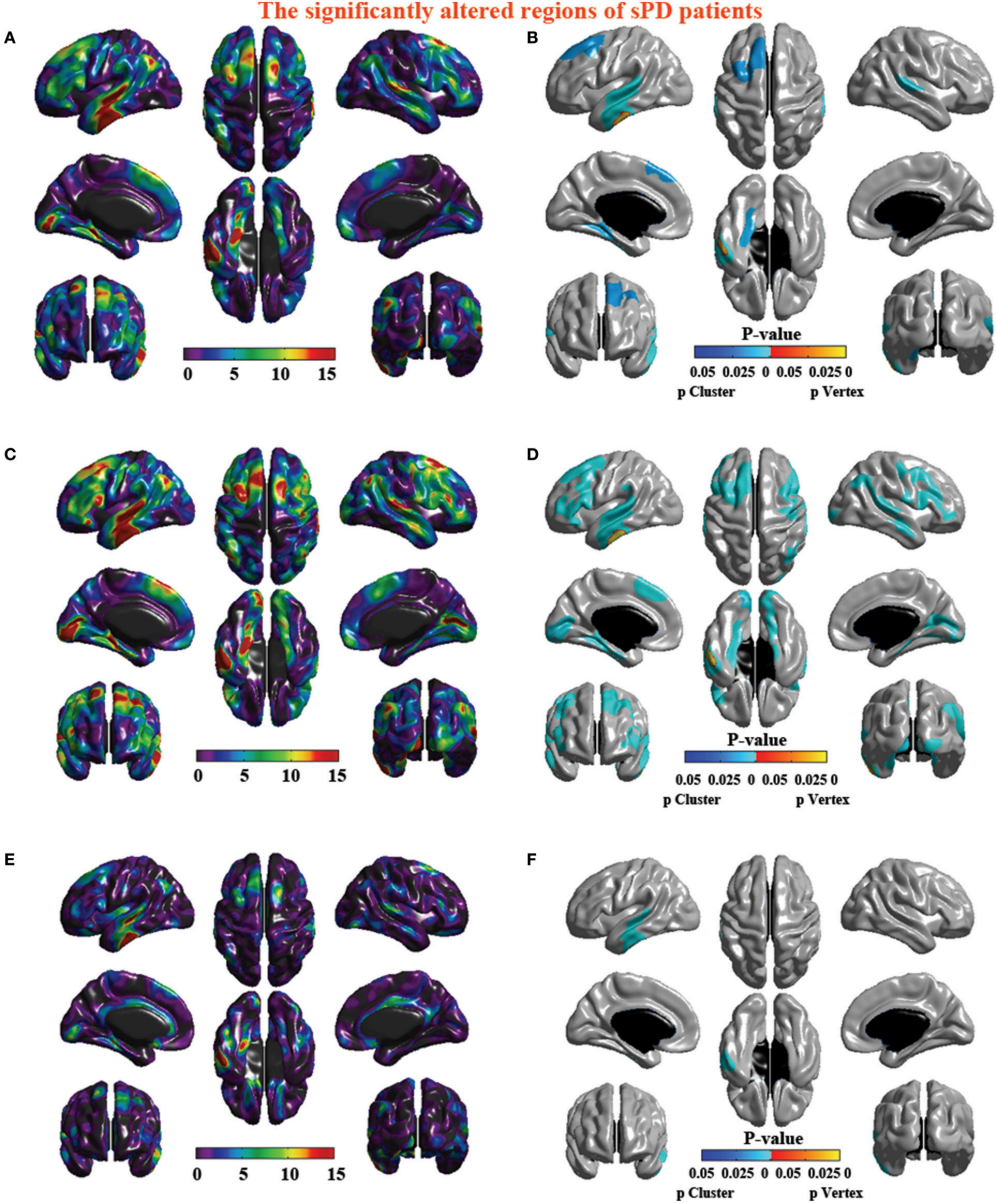
**The significant altered brain regions of cortical thickness in the sPD brain in the analysis of different regression covariates. (A)** The significantly altered brain regions in the F-map in the analysis of the regression of no covariates. **(B)** The significantly altered brain regions after the Family wise error (FWE) correction in the analysis of the regression of no covariates. **(C)** The significantly altered brain regions in the F-map in the analysis of the regression of brain size covariates. **(D)** The significantly altered brain regions after the FWE correction in the analysis of regression of brain size covariates. **(E)** The significantly altered brain regions in the F-map in the analysis of the regression of cortical thickness covariates. **(F)** The significantly altered brain regions after the FWE correction in the analysis of the regression of cortical thickness covariates.

The major results regarding the analysis of local cortical thickness are summarized in Table 3. The local cortical thickness was analyzed using the regression of no covariates (4 clusters), the regression of brain size (7 clusters) and the cortical surface covariates (1 cluster). We found 12 clusters exhibiting a thinning trend of cortical thickness associated with sPD compared with the control group (Table 3, Figures 2–4). The brain regions presented the decreased cortical thickness compared by the regression of no covariates consisted of Heschl-L, Temporal-Sup-L, Temporal-Inf-L, Temporal-Mid-L, Heschl-R, Temporal-Sup-R, SupraMarginal-R, ParaHippocampal-L, Lingual-L, Fusiform-L, Frontal-Sup-L, Supp-Motor-Area-L, Frontal-Mid-L, Frontal-Sup-Medial-L (Figures 2A–H, 4A,B). The brain regions presented the decreased cortical thickness compared by the regression of brain size covariates included Frontal-Sup-R, Frontal-Inf-Oper-R, Frontal-Mid-R, Frontal-Inf-Orb-R, Frontal-Mid-Orb-R, Frontal-Inf-Tri-R, Rolandic-Oper-R, Postcentral-R, Precentral-R, ParaHippocampal-R, Calcarine-R, Lingual-R, Fusiform-R, Precuneus-R, Cuneus-R, Occipital-Sup-R, Occipital-Inf-R, Occipital-Mid-R, Parietal-Inf-R, Angular-R, Heschl-R, Temporal-Sup-R, Temporal-Mid-R, SupraMarginal-R, Occipital-Mid-R, Frontal-Sup-L, Supp-Motor-Area-L, Frontal-Inf-Oper-L, Frontal-Mid-L, Frontal-Sup-Orb-L, Frontal-Inf-Orb-L, Frontal-Mid-Orb-L, Frontal-Sup-Medial-L, Frontal-Inf-Tri-L Precentral-L, ParaHippocampal-L, Lingual-L, Fusiform-L, Precuneus-L, Heschl-L, Temporal-Sup-L, Temporal-Inf-L, Temporal-Mid-L, SupraMarginal-L, Calcarine-L, Lingual-L, Cuneus-L (Figures 3A–N, 4C,D). The brain regions presented the decreased cortical thickness compared by the regression of cortical surface covariates included Temporal-Sup-L, Temporal-Inf-L, Temporal-Mid-L (Figures 3O,P, 4E,F). No cortical regions exhibited the increase thickness in the comparison between sPD and control groups (Figures 2A,C,E,G, 3A,C,E,G,I,K,M,O). Among them, the brain regions that the comparison of the uncorrected average value of cortical thickness in the significant different brain regions between sPD and controls showed the significant decrease were Heschl-L, Temporal-Sup-L, Temporal-Mid-L, Occipital-Mid-R, Precuneus-L, Calcarine-L, Lingual-L, and Cuneus-L (Table 3).

**Table 3:** **Differences between cortical thickness within significant regions between sPD and control**.

	**Coordinates**			**Mean thickness**		**Voxel**	**Peak F score**	***P*-value**
	***X***	***Y***	***Z***	**HC**	**sPD**			
**REGIONS OF INTEREST (REGRESSION OF NO COVARIATES) (sPD<CONTROL)**								
**Cluster 1**								
Heschl-L^*^	−59.1239	−10.9802	1.79163	2.8036 ± 1.365	2.6662 ± 4.478	9	7.7174	0.04479^*^
Temporal-Sup-L^*^	−55.958	−27.4985	0.278152	2.9933 ± 0.8593	2.8286 ± 3.445	637	18.0768	0.010423^*^
Temporal-Inf-L	−54.4454	−15.2431	−33.9239	3.4058 ± 1.8628	3.1782 ± 2.9692	304	27.0206	0.259457
Temporal-Mid-L	−60.003	−20.8681	−22.3413	3.3238 ± 1.0183	3.1488 ± 3.256	769	20.8498	0.052188
**Cluster 2**								
Heschl-R	51.8826	−22.4111	8.10232	2.7391 ± 1.3882	2.5882 ± 3.066	101	16.2814	0.333542
Temporal-Sup-R	53.4681	−24.4854	9.01416	2.8916 ± 1.3334	2.7195 ± 3.812	597	17.8728	0.102827
SupraMarginal-R	52.762	−35.4889	18.4078	2.8026 ± 1.5014	2.6667 ± 4.461	1	7.1233	0.082211
**Cluster 3**								
ParaHippocampal-L	−28.9172	−31.9334	−17.2318	3.1389 ± 1.9343	2.957 ± 2.9414	490	17.4529	0.208735
Lingual-L	−24.6297	−55.8759	−7.8232	2.9769 ± 0.9651	2.8569 ± 1.752	176	12.6554	0.422723
Fusiform-L	−32.5946	−31.8994	−18.0468	3.2451 ± 1.1522	3.1063 ± 2.633	201	14.4344	0.293891
**Cluster 4**								
Frontal-Sup-L	−18.405	27.4339	56.109	3.2483 ± 1.673	3.0918 ± 3.584	487	12.3403	0.365677
Supp-Motor-Area-L	−11.7888	26.6935	57.4478	3.4401 ± 2.5909	3.239 ± 6.7413	204	11.913	0.164918
Frontal-Mid-L	−33.1085	6.79534	56.3678	3.1386 ± 1.6943	2.9817 ± 3.377	316	13.0255	0.460781
Frontal-Sup-Medial-L	−8.68275	33.1477	54.8355	3.5221 ± 2.1019	3.342 ± 4.8471	191	13.0086	0.284553
**REGIONS OF INTEREST (REGRESSION OF BRAIN SIZE COVARIATES) (sPD<CONTROL)**								
**Cluster 1**								
Frontal-Sup-R	29.4901	−10.5107	58.981	2.912 ± 1.3613	2.8188 ± 3.984	10	9.0341	0.088605
Frontal-Inf-Oper-R	45.0591	13.2315	23.6988	3.2341 ± 1.4757	3.138 ± 2.2269	270	11.7553	0.201087
Frontal-Mid-R	42.1644	31.2292	18.8666	3.0873 ± 1.4474	2.9656 ± 3.048	557	12.7874	0.388212
Frontal-Inf-Orb-R	44.5502	43.562	−2.72275	3.2854 ± 1.5231	3.1707 ± 2.405	41	10.885	0.250419
Frontal-Mid-Orb-R	42.7927	46.9668	−1.81597	3.2234 ± 1.2054	3.1203 ± 2.124	60	11.3122	0.374341
Frontal-Inf-Tri-R	43.0222	30.2529	17.4664	3.0905 ± 1.1183	2.9787 ± 2.331	330	14.1156	0.399803
Rolandic-Oper-R	59.5857	−6.24091	12.3292	3.1205 ± 1.8114	3.0124 ± 3.035	47	9.8802	0.318654
Postcentral-R	42.7618	−17.2416	47.954	2.4351 ± 0.9592	2.3367 ± 2.368	353	18.0982	0.210631
Precentral-R	39.9619	−17.0107	47.0979	2.706 ± 1.121	2.6008 ± 3.498	420	16.1202	0.060772
**Cluster 2**								
ParaHippocampal-R	23.4286	−26.9602	−20.2916	3.2363 ± 1.9298	3.0965 ± 4.030	185	11.0541	0.399803
Calcarine-R	9.62688	−77.1555	11.6999	2.6676 ± 1.0073	2.5592 ± 2.993	629	20.3872	0.082211
Lingual-R	21.9604	−56.5697	−7.91298	2.9457 ± 0.8961	2.8425 ± 2.012	396	11.7836	0.313257
Fusiform-R	24.1216	−73.1373	−8.81925	2.9642 ± 1.2979	2.8739 ± 2.312	11	8.0218	0.398133
Precuneus-R	19.7339	−62.5681	16.9486	3.0264 ± 1.5017	2.9401 ± 2.196	51	13.5812	0.172704
Cuneus-R	19.7435	−62.2524	11.4104	2.7444 ± 1.419	2.6405 ± 2.913	364	15.6829	0.423608
Occipital-Sup-R	22.1019	−100.9825	−3.11279	2.5193 ± 2.009	2.3921 ± 3.8834	50	10.4953	0.499524
Occipital-Inf-R	26.692	−98.802	−4.01854	2.5924 ± 1.6237	2.4828 ± 3.136	57	9.5471	0.499524
Occipital-Mid-R	25.2149	−99.6235	−3.75998	2.5933 ± 1.1324	2.5011 ± 2.617	33	9.6531	0.275444
**Cluster 3**								
Parietal-Inf-R	53.0044	−50.2854	42.8408	3.0598 ± 1.8194	2.9675 ± 3.270	5	8.0915	0.398133
Angular-R	46.4373	−66.9658	37.897	3.1241 ± 1.2744	3.0089 ± 2.097	372	13.4322	0.297979
Heschl-R	51.8826	−22.4111	8.10232	2.7577 ± 1.3399	2.6138 ± 3.079	133	20.8527	0.284553
Temporal-Sup-R	53.4681	−24.4854	9.01416	2.9654 ± 1.1474	2.813 ± 3.1992	938	23.3982	0.114864
Temporal-Mid-R	61.9148	−12.7059	−16.1903	3.4737 ± 1.0343	3.3537 ± 2.3797	369	12.3128	0.060772
SupraMarginal-R	51.1909	−35.265	18.3435	2.8329 ± 1.2927	2.7254 ± 4.035	19	10.6551	0.060772
Occipital-Mid-R^*^	43.227	−61.38322	39.2208	3.1584 ± 0.7103	3.0635 ± 2.524	94	11.4343	0.025177^*^
**Cluster 4**								
Frontal-Sup-L	−18.405	27.4339	56.1039	3.2203 ± 1.6142	3.0701 ± 3.451	616	14.4461	0.365677
Supp-Motor-Area-L	−7.94114	22.794	59.941	3.4617 ± 2.5469	3.2852 ± 5.7485	408	14.4189	0.303459
Frontal-Inf-Oper-L	−52.7537	13.7529	14.5111	3.0934 ± 1.1378	2.9876 ± 1.992	216	14.8618	0.374341
Frontal-Mid-L	−29.8748	7.63778	55.0685	3.0966 ± 1.3588	2.9666 ± 2.216	895	16.858	0.287994
Frontal-Sup-Orb-L	−28.0227	54.698	−0.38587	2.9854 ± 1.0589	2.8873 ± 2.536	1	6.9932	0.241275
Frontal-Inf-Orb-L	−42.9491	28.4152	−11.7796	3.2064 ± 1.5181	3.0876 ± 2.974	271	15.2785	0.48645
Frontal-Mid-Orb-L	−42.6875	47.1491	−0.71955	3.0357 ± 1.1788	2.9413 ± 2.327	30	7.9455	0.473533
Frontal-Sup-Medial-L	−8.21667	31.7123	55.4035	3.565 ± 2.196	3.3965 ± 4.366	246	14.6059	0.460781
Frontal-Inf-Tri-L	−52.294	17.85	19.4534	2.9938 ± 1.2021	2.8815 ± 2.2496	549	12.3206	0.460967
Precentral-L	−47.9393	8.81713	19.8343	3.1261 ± 1.2709	3.028 ± 2.1127	20	9.6791	0.3082
**Cluster 5**								
ParaHippocampal-L	−28.9172	−31.9334	−17.2318	3.2524 ± 1.9164	3.0802 ± 2.843	687	18.6054	0.18646
Lingual-L	−22.1588	−58.3478	−8.18995	2.9542 ± 1.0141	2.8386 ± 1.648	279	13.2456	0.278245
Fusiform-L	−32.6619	−30.7991	−18.836	3.2684 ± 1.1662	3.1327 ± 2.550	238	15.5417	0.344028
Precuneus-L^*^	−18.5619	−38.5194	−0.49079	2.8118 ± 2.0257	2.7225 ± 2.379	18	10.1712	0.044989^*^
**Cluster 6**								
Heschl-L^*^	−59.2778	−12.075	2.28861	2.7072 ± 1.0744	2.5958 ± 3.785	91	10.086	0.027191^*^
Temporal-Sup-L^*^	−57.4669	−27.1693	0.847994	2.9608 ± 0.8592	2.8093 ± 3.316	875	19.001	0.013619^*^
Temporal-Inf-L	−58.2652	−24.0822	−26.477	3.3942 ± 1.7706	3.1768 ± 2.8402	336	33.1134	0.268733
Temporal-Mid-L	−59.4378	−19.9477	−23.2011	3.3228 ± 1.0086	3.1503 ± 3.238	811	23.824	0.050234
SupraMarginal-L	−59.795	−31.5237	31.9739	3.0372 ± 1.5212	2.9133 ± 3.400	92	8.437	0.313257
**Cluster 7**								
Calcarine-L^*^	−7.23996	−90.2558	−9.22916	2.6311 ± 0.8214	2.5039 ± 2.676	708	20.8572	0.046537^*^
Lingual-L^*^	−9.67694	−84.8585	−11.3099	2.8113 ± 0.6943	2.703 ± 2.7056	52	14.3806	0.012616^*^
Cuneus-L^*^	−9.64496	−70.097	13.9467	2.44 ± 0.6633	2.3507 ± 2.423	50	12.5979	0.020773^*^
**REGIONS OF INTEREST (REGRESSION OF CORTICAL SURFACE COVARIATES) (sPD<CONTROL)**								
Temporal-Sup-L^*^	−55.5064	−20.9084	−3.59104	3.0092 ± 0.908	2.8283 ± 3.5085	285	15.1268	0.013619^*^
Temporal-Inf-L	−54.4454	−15.2431	−33.9239	3.3968 ± 2.0485	3.1469 ± 3.3749	238	21.742	0.297979
Temporal-Mid-L	−60.003	−20.8681	−22.3413	3.3456 ± 1.2734	3.1469 ± 3.6706	423	15.8321	0.095468

*X, Y, and Z were in MNI coordinates. For each cluster, we report the brain regions of the highest peak value. Thickness is expressed in mm*.

* *Indicates a significance of p ≤ 0.05 uncorrected*.

### Abnormal brain regions of cortical surface in sPD patients vs. control

**Figure 5:**
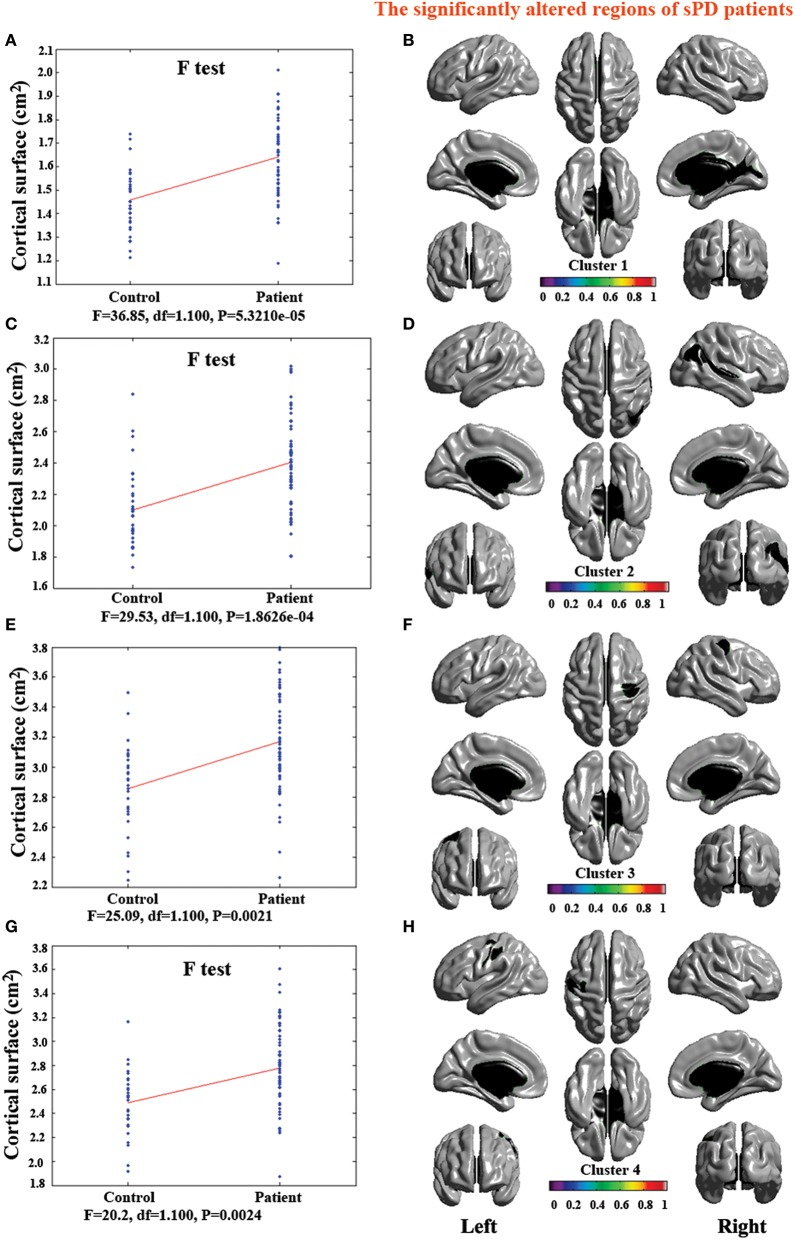
**The comparison of cortical surface between sPD and control using the regression of no covariates. (A,C,E,G)** The *F*-test of the average cortical surface in the cluster 1–4 between sPD and the control. **(B,D,F,H)** The significantly altered brain regions in the cluster 1–4 of the sPD brain. The cortical surface of all brain regions were significantly enlarged in sPD compared with the control in the analysis of the regression of no covariates.

**Figure 6:**
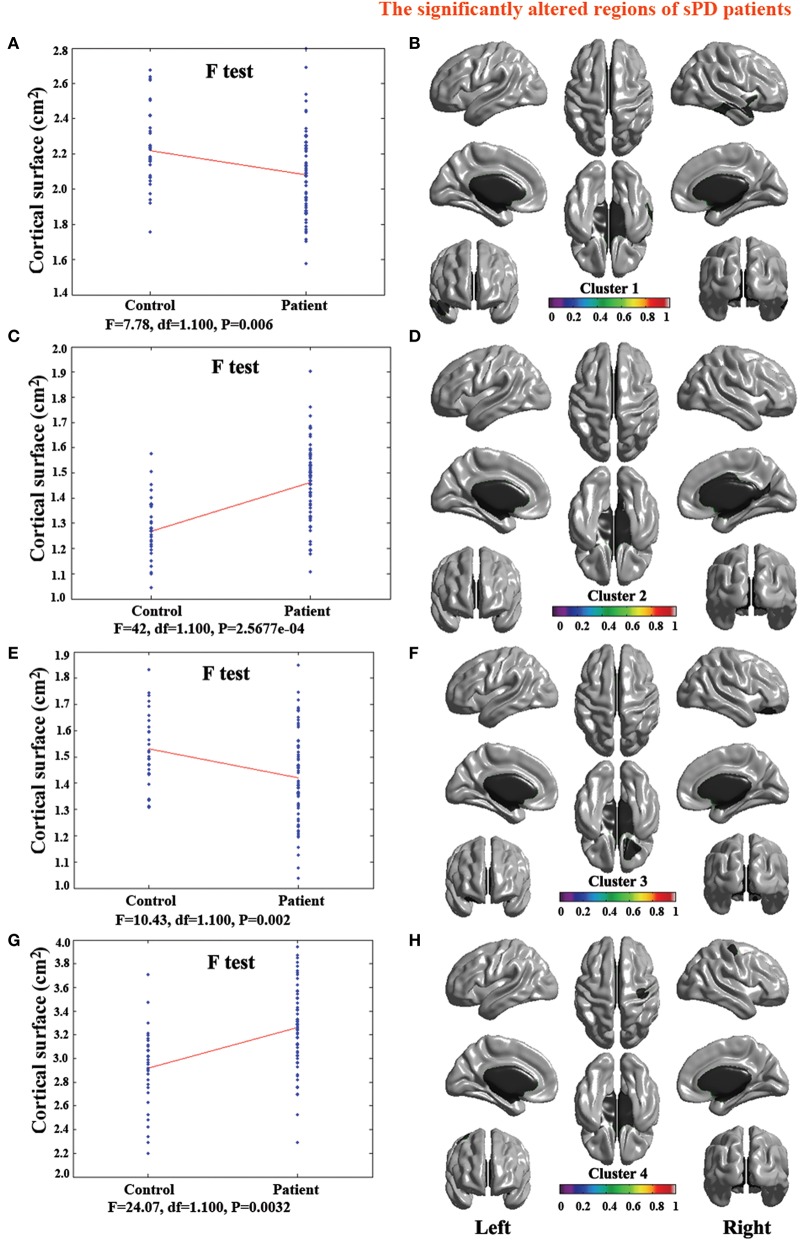
**The comparison of cortical surface between sPD and the control using the regression of brain size covariates. (A,C,E,G)** The *F*-test of the average cortical surface in the cluster 1–4 between sPD and the control. **(B,D,F,H)** The significantly altered brain regions in the cluster 1–4 of the sPD brain. The cortical surface of brain regions in the cluster 1 and 3 were significantly shrunk, that in the cluster 2 and 4 were significantly enlarged in sPD compared with the control in the analysis of the regression of brain size covariates.

**Figure 7:**
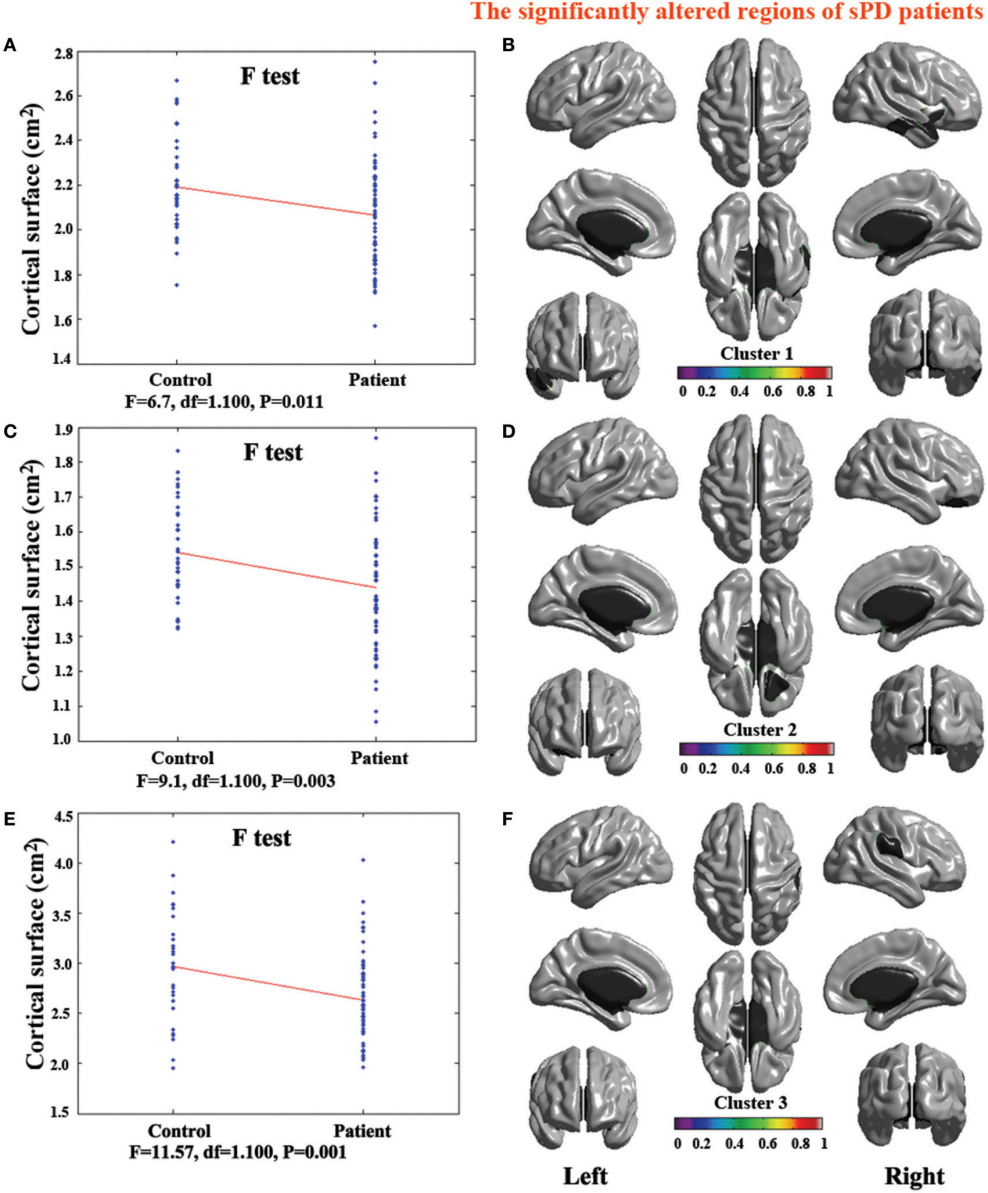
**The comparison of cortical surface between sPD and the control using the regression of cortical surface covariates. (A,C,E)** The *F*-test of the average cortical surface in the cluster 1–3 between sPD and the control. **(B,D,F)** The significantly altered brain regions in the cluster 1–3 of the sPD brain. The cortical surface of all brain regions were significantly shrunk in sPD compared with the control in the analysis of the regression of cortical surface covariates.

**Figure 8:**
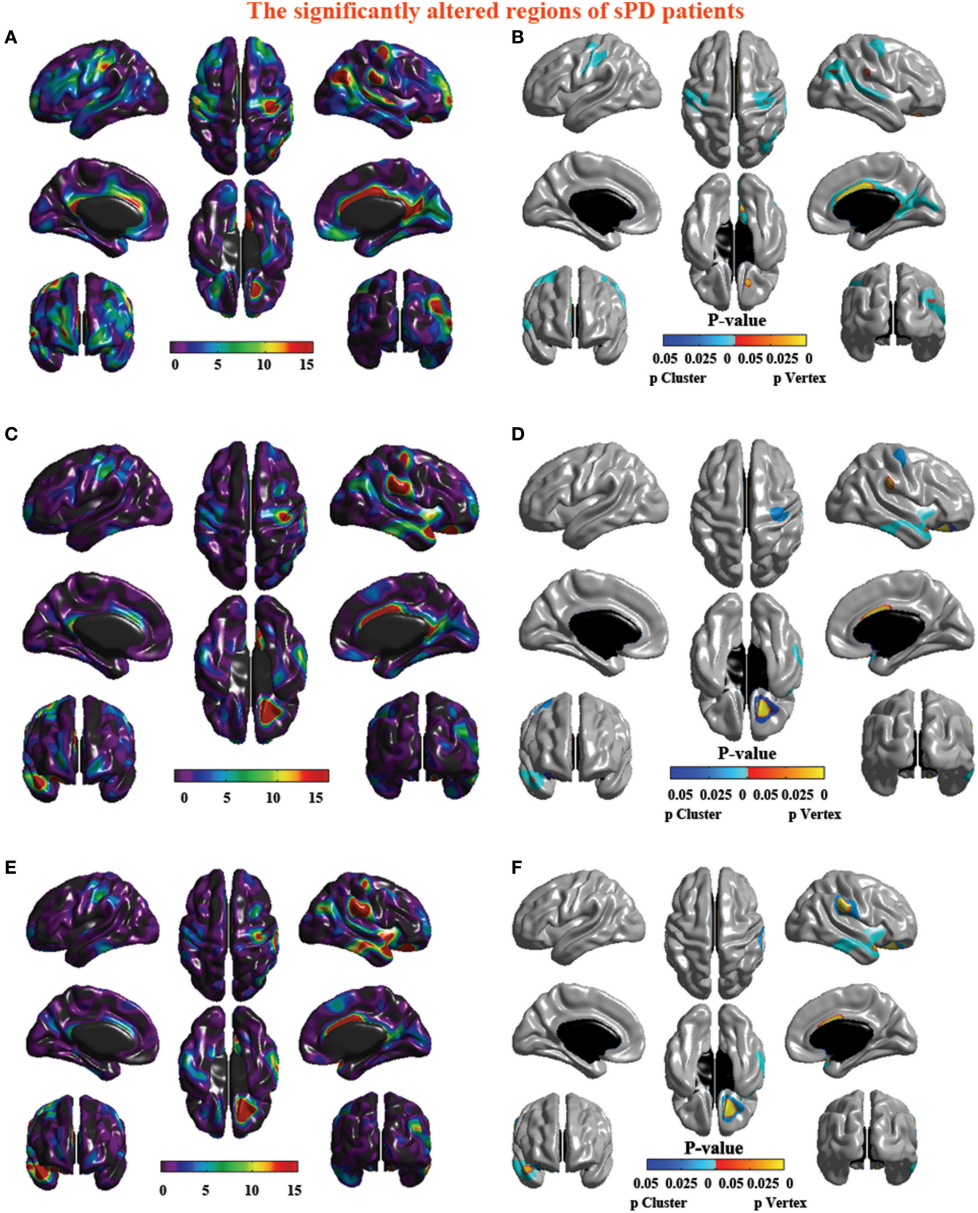
**The significantly altered brain regions of cortical surface in the sPD brain in the analysis of the regression of different covariates. (A)** The significantly altered brain regions in the F-map in the analysis of the regression of no covariates. **(B)** The significantly altered brain regions after the FWE correction in the analysis of the regression of no covariates. **(C)** The significantly altered brain regions in the F-map in the analysis of the regression of brain size covariates. **(D)** The significantly altered brain regions after the FWE correction in the analysis of the regression of brain size covariates. **(E)** The significantly altered brain regions in the F-map in the analysis of the regression of cortical surface covariates. **(F)** The significantly altered brain regions after the FWE correction in the analysis of the regression of cortical surface covariates.

The analysis of cortical surface demonstrated that the sPD patients had several regions with the significant focal cortical enlargement or shrinkage compared to controls applying the regression of different covariates (Table 4, Figures 5–8). We evaluated the cortical surface using the regression of no covariates, the regression of brain size and cortical surface. In the analysis of the regression of no covariates, all cortical surface were enlarged, including Cingulum-Post-R, Calcarine-R, Cingulum-Mid-R, Cingulum-Ant-R, Lingual-R, Precuneus-R, Cuneus-R, Angular-R, Heschl-R, Temporal-Sup-R, Temporal-Mid-R, Occipital-Mid-R, Frontal-Sup-R, Postcentral-R, Precentral-R, Parietal-Inf-L, SupraMarginal-L, Postcentral-L, and Precentral-L (Figures 5A–H, 8A,B). In the analysis of regression of brain size covariates, the shrunk cortical surfaces were in Insula-R, Temporal-Pole-Sup-R, Temporal-Pole-Mid-R, Temporal-Sup-R, Temporal-Inf-R, Temporal-Mid-R, Rectus-R, Frontal-Sup-Orb-R, Frontal-Inf-Orb-R, and Frontal-Mid-Orb-R (Figures 6A,B,E,F, 8C,D); The enlarged cortical surfaces were in Cingulum-Post-R, Cingulum-Mid-R, Cingulum-Ant-R, Precuneus-R, Cuneus-R, Postcentral-R and Precentral-R (Figures 6C,D,G,H, 8C,D). In the analysis of the regression of cortical surface covariates, all cortical surface were shrunk, including Insula-R, Temporal-Pole-Sup-R, Temporal-Pole-Mid-R, Temporal-Sup-R, Temporal-Inf-R, Temporal-Mid-R, Rectus-R, Frontal-Sup-Orb-R, Frontal-Inf-Orb-R, Frontal-Mid-Orb-R, SupraMarginal-R, and Postcentral-R (Figures 7A–F, 8E,F). Among them, the brain regions that the comparison of the uncorrected average value of cortical surface in the significant different brain regions between sPD and controls showed the significant increase were Cingulum-Mid-R, Temporal-Sup-R, Parietal-Inf-L, Postcentral-L, and Precuneus-R, the significant decrease were Frontal-Inf-Orb-R, Frontal-Mid-Orb-R and Temporal-Inf-R (Table 4).

**Table 4:** **Differences of the cortical surface within significant regions between sPD and control**.

	**Coordinates**			**Mean thickness**		**Voxel**	**Peak F score**	***P*-value**
	***X***	***Y***	***Z***	**HC**	**sPD**			
**REGIONS OF INTEREST (REGRESSION OF NO COVARIATES) (sPD vs. CONTROL)**								
**Cluster 1**								
Cingulum-Post-R	6.06224	−47.045	11.5543	1.3919 ± 1.1864	1.5407 ± 3.3651	171	20.1449	0.106703
Calcarine-R	5.78068	−86.5958	8.72105	2.6572 ± 6.4073	2.9574 ± 15.2174	165	8.9333	0.249481
Cingulum-Mid-R^*^	3.90541	2.52516	30.2404	1.5224 ± 0.8512	1.731 ± 2.9829	469	39.1245	0.028257^*^
Cingulum-Ant-R	3.90898	6.33644	29.068	1.3695 ± 1.7217	1.5914 ± 3.5693	304	38.6549	0.411603
Lingual-R	16.5966	−44.2568	−2.90858	1.0247 ± 1.0204	1.1263 ± 2.048	59	8.5379	0.448204
Precuneus-R	11.5642	−60.8899	21.2005	1.1778 ± 0.5257	1.3277 ± 1.6647	879	24.6867	0.056322
Cuneus-R	15.8779	−56.3339	12.899	1.6265 ± 1.922	1.8123 ± 3.5413	474	15.9316	0.435298
**Cluster 2**								
Angular-R	49.4232	−66.2377	32.6919	2.3456 ± 5.8295	2.7331 ± 18.1756	206	18.0348	0.060772
Heschl-R	59.1239	−10.9802	1.79163	1.9612 ± 2.7929	2.1541 ± 5.2657	46	14.5007	0.460967
Temporal-Sup-R^*^	58.939	−9.86726	1.2665	2.0558 ± 1.224	2.2829 ± 4.2417	686	14.6511	0.030515^*^
Temporal-Mid-R	48.0109	−68.3659	29.871	1.781 ± 4.2261	2.1069 ± 12.8331	337	19.8414	0.070716
Occipital-Mid-R	45.31	−70.9401	30.147	2.3373 ± 6.6996	2.7428 ± 17.5088	381	20.0935	0.159153
**Cluster 3**								
Frontal-Sup-R	29.5585	−11.3362	60.6738	2.9542 ± 8.8537	3.3083 ± 25.059	27	10.0596	0.106703
Postcentral-R	40.4793	−21.9485	54.2606	2.2604 ± 1.8104	2.474 ± 4.1709	247	19.6211	0.284553
Precentral-R	39.6868	−21.6645	54.2762	3.2701 ± 4.4214	3.6517 ± 10.2824	349	19.5012	0.275444
**Cluster 4**								
Parietal-Inf-L^*^	−45.7169	−27.8002	41.7718	2.284 ± 5.3381	2.5975 ± 17.4042	59	11.8819	0.046537^*^
SupraMarginal-L	−58.2636	−20.3987	35.4759	2.4962 ± 7.7406	2.7893 ± 20.1359	2	7.0805	0.164918
Postcentral-L^*^	−48.7247	−24.6917	42.5797	2.3336 ± 2.4283	2.6122 ± 8.0792	686	13.6881	0.039927^*^
Precentral-L	−37.7797	−21.3469	59.8623	3.2245 ± 4.8763	3.5455 ± 12.9001	164	9.9391	0.153567
**REGRESSION OF BRAIN SIZE COVARIATES**								
**Cluster 1**								
Insula-R	37.0457	11.459	−5.11724	1.6581 ± 1.1291	1.5974 ± 1.757	360	15.3011	0.233048
Temporal-Pole-Sup-R	48.001	13.373	−21.2642	2.2727 ± 3.573	2.1129 ± 6.76	340	21.0941	0.47404
Temporal-Pole-Mid-R	49.2513	9.34588	−27.3505	1.819 ± 3.6959	1.7182 ± 6.9567	23	12.7017	0.460967
Temporal-Sup-R	45.6246	4.48984	−14.9187	1.449 ± 1.0952	1.374 ± 2.0063	124	14.9705	0.435298
Temporal-Inf-R	60.4325	−24.4156	−20.3328	3.75 ± 11.4339	3.4791 ± 35.1107	116	9.9928	0.068093
Temporal-Mid-R	60.7466	−13.3493	−14.8402	2.5956 ± 4.8873	2.4116 ± 9.9658	298	11.6357	0.423608
**Cluster 2**								
Cingulum-Post-R	6.06224	−47.045	11.5543	0.9428 ± 0.3758	1.0465 ± 1.1769	56	13.5429	0.058506
Cingulum-Mid-R^*^	3.90541	2.52516	30.2404	1.6102 ± 0.9186	1.8604 ± 3.855	280	31.3582	0.007129^*^
Cingulum-Ant-R	3.90898	6.33644	29.068	1.3935 ± 1.888	1.6503 ± 4.1851	211	30.8332	0.323285
Precuneus-R^*^	10.6212	−61.2469	21.4594	1.1278 ± 0.5454	1.285 ± 1.8178	611	18.5368	0.039927^*^
Cuneus-R	15.8779	−56.3339	12.899	1.1084 ± 1.049	1.2635 ± 2.5485	103	9.7254	0.225522
**Cluster 3**								
Rectus-R	12.2748	25.4372	−20.2742	1.0897 ± 0.6708	1.0534 ± 1.202	113	29.5018	0.398133
Frontal-Sup-Orb-R	15.3925	27.5656	−24.5147	1.6363 ± 0.9628	1.4975 ± 2.2645	449	53.3	0.25791
Frontal-Inf-Orb-R	21.3728	25.5892	−20.3636	1.5475 ± 0.8878	1.4606 ± 2.8211	215	27.9476	0.054217
Frontal-Mid-Orb-R	26.5133	38.58	−14.4089	1.1117 ± 0.4827	1.0692 ± 1.544	2	9.3197	0.052188
**Cluster 4**								
Postcentral-R	40.4793	−21.9485	54.2606	2.2259 ± 2.087	2.4557 ± 4.3658	126	14.0816	0.399803
Precentral-R	37.2299	−18.1214	63.0762	3.3336 ± 5.4596	2.4557 ± 4.3658	209	13.9398	0.293891
**REGRESSION OF CORTICAL SURFACE COVARIATES**								
**Cluster 1**								
Insula-R	39.1071	7.22324	−18.5732	1.6007 ± 1.0403	1.549 ± 1.6686	503	20.9358	0.268733
Temporal-Pole-Sup-R	48.001	13.373	−21.2642	2.2928 ± 3.4192	2.1431 ± 6.8075	371	24.4722	0.460781
Temporal-Pole-Mid-R	49.2513	9.34588	−27.3505	2.0641 ± 4.479	1.9722 ± 9.5039	36	16.3836	0.376835
Temporal-Sup-R	43.1269	2.1696	−15.5755	1.5393 ± 1.2161	1.4659 ± 2.3506	180	19.038	0.499524
Temporal-Inf-R	60.4325	−24.4156	−20.3328	3.4973 ± 8.8036	3.262 ± 28.2562	173	12.7743	0.050234
Temporal-Mid-R	60.7466	−13.3493	−14.8402	2.6117 ± 4.8373	2.4376 ± 9.5722	372	14.2843	0.473533
**Cluster 2**								
Rectus-R	12.2748	25.4372	−20.2742	1.1118 ± 0.6896	1.0785 ± 1.2177	136	32.1403	0.386133
Frontal-Sup-Orb-R	14.4781	28.0252	−24.6949	1.6343 ± 0.9571	1.4996 ± 2.2674	473	57.1807	0.249481
Frontal-Inf-Orb-R	22.9301	28.3291	−18.3183	1.6076 ± 0.9529	1.5269 ± 2.998	256	30.1994	0.058506
Frontal-Mid-Orb-R^*^	26.5133	38.58	−14.4089	1.1878 ± 0.5771	1.1521 ± 1.8743	6	12.5006	0.046537^*^
**Cluster 3**								
SupraMarginal-R	60.5612	−30.4401	31.9716	2.9363 ± 12.2883	2.5444 ± 15.1193	540	34.0177	0.061332
Postcentral-R	59.8486	−19.17	24.5333	3.049 ± 7.1149	2.8657 ± 13.707	195	19.5353	0.499524

*X, Y, and Z were in MNI coordinates. For each cluster, we report the highest peak value. Cortical surface is expressed in cm^2^*.

* *Indicates a significance of p ≤ 0.05 uncorrected*.

## Discussion

In this study, the cortical morphometric alterations including the volume, thickness, surface, and density in both the intermediate sPD patients and the controls from HPCM were detected and compared, the cortical densities were not different, which result was corresponded to previously published studies about the alteration of cortical density in sPD (Feldmann et al., 2008b). The cortical volumes were significantly diminished, the cortical thickness was significantly thinned, the cortical surfaces exhibited either significantly expansion or shrinkage associated with the disease in the patients with sPD compared with the controls.

The results presented here revealed that the alteration of cortical surface were not completely accordant to the previous reports. In our result, the sPD patients exhibited an enlarged cortical surface in the analysis of the no regression and the regression covariates of cortical surface, but showed a surface reduction in the partial cortical regions of the bilateral hemisphere in the sPD patients while analyzed the cortical surface using the brain size for regression covariates. This could seem be contradictory with the previous results, but we suggested that the cortical surface was closely related to the geometric structure of brain. The cortical surface analysis of simple VBM studies only base on a flat surface of cortex, however, the method of cortical morphometry allows us to eliminate the geometric affect of the cortical volume and thickness alteration. The cortical surface might mainly be related to the degree of local cortical folding, because the atrophy of cortex could result in more tension or shrinkage of sulci and extended or reduced the cortical surface. In order to identify the possibility, we used the brain size for regression covariates to further analyze the alteration of cortical surface, found that the cortical surfaces in the certain regions were enlarged, while that in some regions were reduced, it was not that all cortical surfaces were enlarged. It was possible that the atrophy of cortex did not completely enlarge the cortical surfaces in all brain regions, the local brain size was its important effect on the cortical surfaces. When analyzing the cortical surface alteration, using the brain size for regression covariates might more accurately reflect the alteration of cortical surfaces.

sPD is traditionally supposed to be generally associated with the degeneration of substantia nigra, an array of motor dysfunction in the sPD patients' main symptoms was previously suggested to originate from the disruption of substantia nigra-striatal loops contributed to the destruction of dopaminergic neurons in the substantia nigra, but the present studied results suggested that sPD not only damages the substantia nigra-striatum corpus dopamine system, but also involves in the other neural systems besides of the dopaminergic system, including the memory, mental, emotional, sympathetic, parasympathetic, serotonergic and noradrenergic systems, its damage almost affected the whole brain (Braak and Braak, 2000). In this study, we revealed the significant differences between the sPD patients and the controls in the cortical volume, thickness and surface in the analysis of the no regression and the regression using the brain size and the cortical surface for covariates respectively. The extensive cortical volume loss, thickness thinning and surface expansion or shrinkage were shown in a lot of brain regions of the sPD patients compared with the controls, among them, including many local regions of bilateral frontal, temporal, parietal and occipital lobe, limbic system, cerebellum, caudate, and thalamus (Tables 2, 3, Figures 1–8). It suggested that the extensive atrophy of cortex such as cortical volume reduction, thickness thinning and surface enlargement or shrinkage occurred in the intermediate sPD from HPCM, these alterations in the cortical volume, thickness and surface were asymmetric in both hemispheres; i.e., the cortical volume, thickness and surface were significantly changed in the left hemisphere, indicating a correlation with the asymmetric complex clinical manifestations with the multisystem effects in sPD patients, which might be related to the different symptom-dominant side, because the majority of symptom-dominant side in our subjects was in the left side (Table 1).

The neuropathological evidences showed that the neuronal Lewy body formation, apoptosis, necrosis, gliosis and the cortical atrophy gradually occurred in the frontal, parietal, occipital, temporal lobes and limbic system, which resulted in a series of complex clinical manifestations, such as non-motor systems (Braak and Braak, 2000). The occurrence of extensive cortical atrophy possibly involves in the pathological progression of sPD patients. In addition, several previous studies have also demonstrated that the cortical atrophy occurred in the bilateral frontal, temporal, parietal, occipital lobe (Hu et al., 2000; Hosokai et al., 2009; Nobili et al., 2009).

A lot of data suggests that the extensive cortical atrophy in brain might be a substrate for the pathology of sPD (Hu et al., 2000; Hosokai et al., 2009; Nobili et al., 2009), because the complex and varied clinical manifestations of sPD could not be explained by the solely degeneration of substantia nigra-striatal loops, should be associated with more extensively damaged brain regions. Meanwhile, in our study, the brain regions of extensively cortical morphometric alteration in the sPD patients were observed like the previous studies. Thus, we concluded that the extensive brain morphological alteration in the sPD patients resulted in the pathological mechanism of complex clinical manifestations in intermediate sPD from HPCM.

The results in this study showed that the extensive cortical volume reduction was preserved in an intermediate sPD population from HPCM compared with a healthy population. we found several local regions exhibiting cortical volume reduction associated with the disease in Frontal lobe (Sup-, Mid (R)-, Inf-Orb, Inf-Oper, Sup-, Mid-, Sup-Medial-L, Rectus-L,-R, Precentral-L); Temporal lobe (Pole-Sup-L,-R, Inf-L, Calcarine-R, Fusiform-L,-R, Olfactory-L,-R); Parietal lobe (Postcentral-L, Precuneus-L,-R); Occipital lobe (Mid-R, Inf-R, Lingual-L,-R); limbic lobe (Hippocampus-L,-R, Insula-L, Amygdala-L,-R, Cingulum-Ant,-Mid,-Post-L,-R, ParaHippocampal-L,-R, Calcarine-L); Cerebellum (Crus1-L,-R, Crus2-L,-R, -4,-5,-6,-7b,-9,-L,-R, Vermis-4,-5,-6,-7,-8,-9,-10), Caudate-L,-R, Thalamus-L,-R gyrus. Among them, the most potential significant different brain regions were Frontal lobe (Sup-Orb-L, Mid-Orb-L,-R Inf-Orb-L, Sup-L, Mid-L, Sup-Medial-L, Rectus-L,-R); Temporal lobe (Inf-L, Pole-Sup-R, Fusiform-L, Olfactory-L); Parietal lobe (Precuneus-L,-R); Occipital lobe (Lingual-L); limbic lobe (Hippocampus-L,-R, Insula-L, Cingulum-Ant,-Mid, Post-L,-R); Cerebellum (Crus1-L,-R, 2-R, -4,-5-L, -6,-9-L,-R, Vermis-6,-7,-8) gyrus (Table 2, Figure 1).

Meanwhile, the results of this study also revealed that the cortical thickness thinning was preserved in the intermediate sPD from HPCM compared with a healthy population, indicated the local cortical thickness associated with the disease. A cortical thickness thinning trend was observed in the following brain regions: Frontal lobe (Mid-L,-R, Sup-Medial-L, Sup-L,-R, Inf-Oper-L,-R, Sup-Orb-L, Inf-,Mid-Orb-L,-R, Inf-Tri-L,-R, Supp-Motor-Area-L, Rolandic-Oper-R, Precentral-L,-R,); Temporal (Sup-L,-R, Inf-L, Mid-L,-R, Heschl-L,-R); Parietal lobe (Inf-R, Postcentral-R, SupraMarginal-L,-R, Angular-R, Precuneus-L); Occipital lobe (Sup-R, Inf-R, Mid-R, Lingual-L,-R, Fusiform-L,-R, Calcarine-L,-R, Precuneus-R, Cuneus-L,-R,); Limbic lobe (ParaHippocampal-L,-R) (Figures 2–4). This set of results implies a consistent pattern of cortical thickness thinning associated to the disease compared to a healthy population. Among them, the most potential significant different brain regions were Heschl-L, Temporal-Sup-L, Temporal-Mid-L, Occipital-Mid-R, Precuneus-L, Calcarine-L, Lingual-L, and Cuneus-L (Table 3).

Furthermore, the results presented here suggested that the cortical surface in certain regions exhibited a larger cortical surface in sPD patients, but that in some regions showed a shrunk cortical surface in the analysis of different regression covariates, which might be associated with the inhomogeneous alteration of local cortical volume and thickness contributed to the inhomogeneous alteration of the local cortical surface, because we must here consider the geometric implications of this observation, the volume shrinkage and the thickness thinning of local cortex could lead to deeper or shallower sulci and extended or reduced the cortical surface. The regions of cortical surface expansion were in Frontal lobe (Sup-R, Precentral-L-R,); Temporal lobe (Sup-R, Heschl-R, Temporal-Mid-R); Parietal lobe (Inf-L, SupraMarginal-L, Precuneus-R, Postcentral-L,-R, Angular-R); Occipital lobe (Mid-R, Postcentral-R, Calcarine-R, Lingual-R); Limbic lobe (Cingulum-Post-R, Mid-R, Ant-R, Cuneus-R; Figures 5, 6, 8A–D). The shrunk cortical surfaces were in Frontal lobe (Sup-Orb-R, Inf-Orb-R, Mid-Orb-R); Temporal lobe (Pole-Sup-R, Pole-Mid-R, Sup-R, Inf-R, Mid-R, Rectus-R, Insula-R); Parietal lobe (SupraMarginal-R, Postcentral-R) (Figures 7A–F, 8E,F). Among them, the most potential significantly different brain regions were that the significant increase regions were Cingulum-Mid-R, Temporal-Sup-R, Parietal-Inf-L, Postcentral-L and Precuneus-R, that the significant decrease regions were Frontal-Inf-Orb-R, Frontal-Mid-Orb-R and Temporal-Inf-R (Table 4).

The lesion of the above described brain regions could contribute to the motor symptoms including tremor (Caudate), rigidity (Frontal, Parietal cortex), bradykinesia (Frontal and parietal cortex), and postural instability (Frontal, Parietal and cerebellum cortex), and no motor symptoms including an array of neural functional disorders such as speech, cognition, mood, behavior, thought, sleep, the autonomic nervous system, the constipation dysfunction, the gastric dysmotility, several eye, and vision abnormalities, an impaired sense of smell, a sensation of pain, paresthesia and so on (Supplemental Table 1).

The alteration of volume, thickness and surface in cortex are extensive and closely associated with the complex clinical manifestations such as motor, sensory, speech, cognition, mood, behavior, thought, sleep, the autonomic nervous system, the constipation dysfunction, the gastric dysmotility, several eye and vision abnormalities, an impaired sense of smell, a sensation of pain, paresthesia the emotion-behavior, the olfactory sense, the visual sense, the cognitive executive disturbances, the emotion and motivation generation, the internal organ activity, the learning and memory formation, the sleep and wakefulness, and so on in sPD (Supplemental Table 1). The major function of the changed regions of cortical volume, thickness and surface in intermediate sPD from HPCM is strongly related to the generation of the complex clinical manifestations of sPD (Supplemental Table 1). The loss (atrophy) of extensive cortex may contribute to the complex clinical manifestations in our patients, which suggests that a series of motor and no motor symptoms in sPD patients may derived from the impairment of different brain regions in the intermediate stage (Supplemental Table 1).

## Conclusion

In summary, we found that an extensive loss of cortex (Cortical volume reduction, thickness thinning, surface enlargement, or shrinkage) in the intermediate sPD patients from HPCM resulted in the dysfunction of the corresponding brain regions, generating a series of complex clinical manifestations being consistent with the clinical characteristics of sPD (Supplemental Table 1, Table 1). In addition, this study also provided some neuroimage evidences for *in vivo* observing the distributed features of abnormal cortical alteration in sPD, and might provide a further understand of the association between the brain morphological abnormalities and the clinical manifestations in sPD, as well as some potential pathological lesion of sPD, and some objective evidences for the diagnosis of sPD.

## Author contributions

Conceived and designed the experiments: RX, XD. Performed the experiments: XD, MZ, CT, LZ, JZ, ZX, XX, and HG. Analyzed the data: XD, MZ, CT, LZ, JZ, and ZX. Contributed reagents/materials/analysis tools: RX, XD. Wrote the paper: RX, XD. Figures processing: XD, RX. The joint first author and contributed equally to the work: XD, MZ, CT, JZ, LZ, and ZX. The corresponding author: RX. All authors have been involved in the drafting, critical revision and final approval of the manuscript for publication. All authors agree to be accountable for all aspectsof the work in ensuring that questions related to the accuracy or integrity of any part of the work are appropriately investigated and resolved.

## Funding

This work was supported by grants from the National Natural Science Foundation of China (30560042, 81160161, 81360198), the education department of Jiangxi province (GJJ10303) and Jiangxi provincial department of science and technology ([2014]-47).

### Conflict of interest statement

The authors declare that the research was conducted in the absence of any commercial or financial relationships that could be construed as a potential conflict of interest.

## Supplementary material

Supplementary Figure 1 **The illustration diagram of studied procedure**.

Supplementary Table 1 **The possible association between the symptoms of sPD and the morphometric alteration of gray matter**.

## Abbreviations

All abbreviations of brain region name derived from the Anatomical Automatic Labeling (AAL) of Montreal Neurological Institute.
