# Supplementary figures and images for: The Alterations of Cortical Volume, Thickness, Surface, and Density in the Intermediate Sporadic Parkinson's Disease from the Han Population of Mainland China
Source: Front Aging Neurosci. 2016 Aug 3;8:185. doi: 10.3389/fnagi.2016.00185 (PMC4971022; doi:10.3389/fnagi.2016.00185)

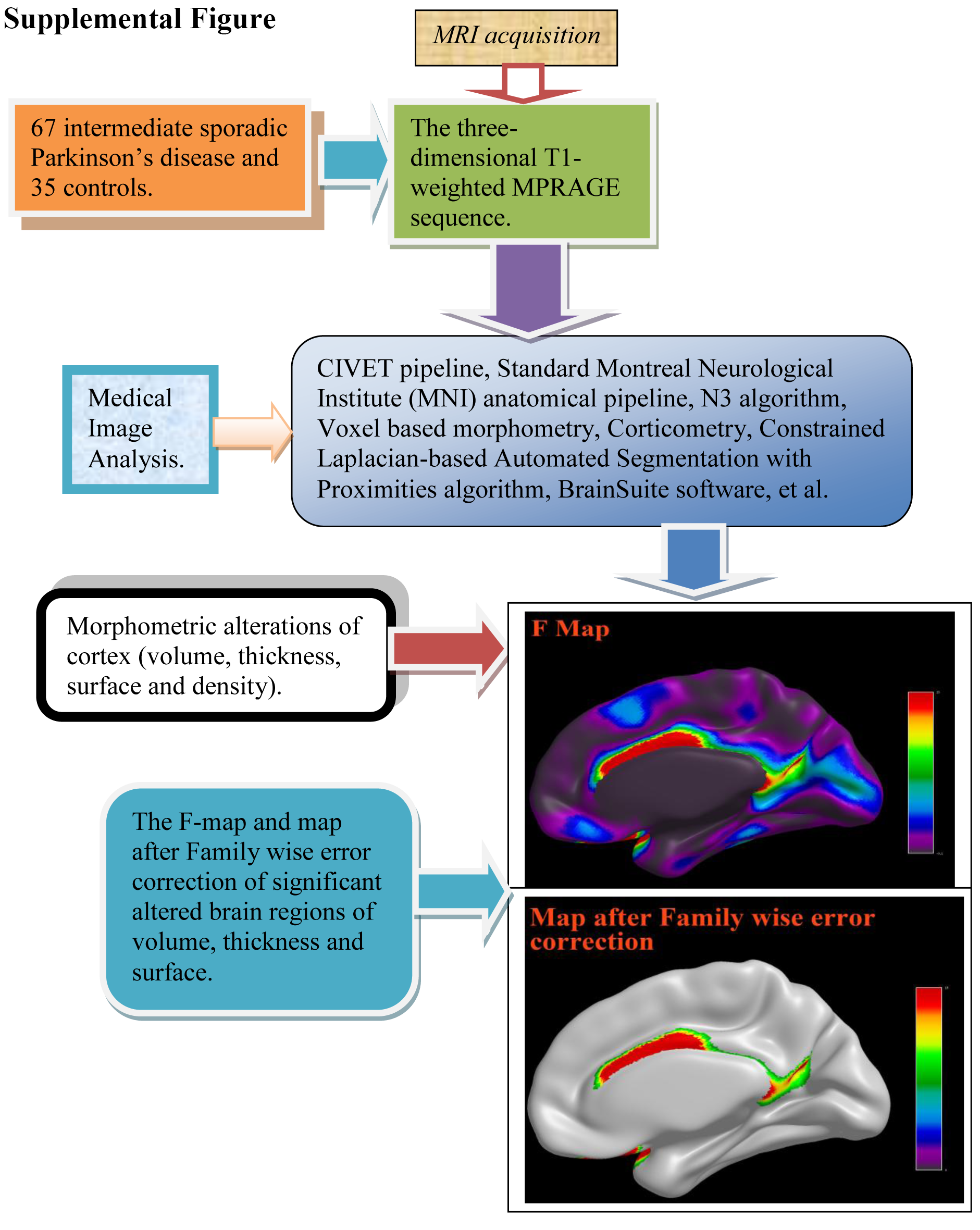

Supplement: Supplementary Figure 1 — The illustration diagram of studied procedure. [file Image1.TIF]
