# Supplementary material for: The Alterations of Cortical Volume, Thickness, Surface, and Density in the Intermediate Sporadic Parkinson's Disease from the Han Population of Mainland China
Source: Front Aging Neurosci. 2016 Aug 3;8:185. doi: 10.3389/fnagi.2016.00185 (PMC4971022; doi:10.3389/fnagi.2016.00185)
Supplement: Supplementary Table 1 — The possible association between the symptoms of sPD and the morphometric alteration of gray matter. [file Table1.DOCX]

Supplemental Table The possible association between the symptoms of sPD and the morphometric alteration of gray matter

| Brain regions of morphometric alteration based on AAL. | Functions of morphometric alterated brain regions. | Related symptoms of sPD possibly derived from the dysfunction of the brain region. |
| --- | --- | --- |
| **Frontal lobe** |  |  |
| **Brain regions of volume reduction** |  |  |
| Frontal-Sup-O[rb](http://en.wikipedia.org/wiki/Orbital_part_of_inferior_frontal_gyrus) -L | Frontal eye fields, saccadic eye movements, thought, cognition, movement planning. | PD is related to several eye and vision abnormalities such as decreased [blink](http://en.wikipedia.org/wiki/Blink) rate, [dry eyes](http://en.wikipedia.org/wiki/Dry_eye), deficient [ocular pursuit](http://en.wikipedia.org/wiki/Smooth_pursuit) (eye tracking) and [saccadic movements](http://en.wikipedia.org/wiki/Saccade) (fast automatic movements of both eyes in the same direction), difficulties in directing gaze upward, and [blurred](http://en.wikipedia.org/wiki/Blurred_vision) or [double vision](http://en.wikipedia.org/wiki/Double_vision). Disorders of cognition, mood, and thought, [Hypokinesia](http://en.wikipedia.org/wiki/Hypokinesia) (slowness of movement) difficulties along the whole course of the movement process, from planning to initiation and finally execution of a movement. Performance of sequential and simultaneous movement is hindered. |
| Frontal-Mid-Orb-L,-R |  |  |
| Frontal-Inf-Orb-L |  |  |
| Frontal-Sup-L | [Self-awareness](http://en.wikipedia.org/wiki/Self-awareness) in coordination with the action of the [sensory system](http://en.wikipedia.org/wiki/Sensory_system), laughter. | Sensation of pain and [paresthesia](http://en.wikipedia.org/wiki/Paresthesia) (skin tingling and numbness), [mask-like face expression](http://en.wikipedia.org/wiki/Hypomimia). |
| Frontal-Sup-Medial-L |  |  |
| Frontal-Mid-L |  |  |
| Frontal-Inf-[Oper](http://en.wikipedia.org/wiki/Opercular_part_of_inferior_frontal_gyrus)-L | Gustatory cortex, taste, movement planning, planning behavior, speech, thought, cognition. | Impaired sense of taste, [hypokinesia](http://en.wikipedia.org/wiki/Hypokinesia), disorders of speech including [voice disorders](http://en.wikipedia.org/wiki/Dysphonia) and aphasia, cognition including [executive dysfunction](http://en.wikipedia.org/wiki/Executive_dysfunction), which can include problems with planning, cognitive flexibility, abstract thinking, rule acquisition, initiating appropriate actions and inhibiting inappropriate actions, and selecting relevant sensory information., mood, behavior, thought. |
| Rectus-L,-R | Sexual desire | [Altered sexual function](http://en.wikipedia.org/wiki/Sexual_dysfunction) |
| Precentral-L | Primary motor cortex, voluntary movement control of different body parts. | Hypokinesia, performance of sequential and simultaneous movement is hindered. [rigidity](http://en.wikipedia.org/wiki/Hypokinesia). |
| **Brain regions of thickness thinning** |  |  |
| Frontal-Sup-L,-R | Same to above | Same to above |
| Supp-Motor-Area-L | Involved in planning complex movements and in co-ordinating movements involving both hands. | Hypokinesia as writing as [small handwriting](http://en.wikipedia.org/wiki/Micrographia_(handwriting)), sewing or getting dressed. |
| Frontal-Mid-L,-R | Thought, cognition, planning behavior, aspects of eye movement control | Disorders of cognition, behavior, and thought. Deficient [ocular pursuit](http://en.wikipedia.org/wiki/Smooth_pursuit) and [saccadic movements](http://en.wikipedia.org/wiki/Saccade), difficulties in directing gaze upward, and [blurred](http://en.wikipedia.org/wiki/Blurred_vision) or [double vision](http://en.wikipedia.org/wiki/Double_vision). |
| Frontal-Sup-Medial-L | Same to above | Same to above |
| Frontal-Sup-Orb-L | Same to above | Same to above |
| Frontal-Inf-Orb-L,-R |  |  |
| Frontal-Mid-Orb-L,-R |  |  |
| Rolandic-Oper-R | Comprises [Broca's area](http://en.wikipedia.org/wiki/Broca%27s_area), involved in semantic tasks, phonological and syntactic processing, [music perception](http://en.wikipedia.org/wiki/Music_cognition), selective response suppression in go/no-go tasks, the suppression of response tendencies related to hand movements. | Disorders of speech as [voice disorders](http://en.wikipedia.org/wiki/Dysphonia). Hypokinesia as writing ([Small handwriting](http://en.wikipedia.org/wiki/Micrographia_(handwriting))), sewing or getting dressed. [festination](http://en.wikipedia.org/wiki/Parkinsonian_gait) (Rapid shuffling steps and a [forward-flexed posture](http://en.wikipedia.org/wiki/Camptocormia) when walking). |
| Frontal-Inf-Oper-L,-R |  |  |
| Frontal-Inf-Tri-L,-R | Comprises [Broca's area](http://en.wikipedia.org/wiki/Broca%27s_area), involved in semantic tasks, such as semantic decision tasks (Determining whether a word represents an abstract or a concrete entity) and generation tasks (Generating a [verb](http://en.wikipedia.org/wiki/Verb) associated with a [noun](http://en.wikipedia.org/wiki/Noun)). | Disorders of speech such as [expressive aphasia](http://en.wikipedia.org/wiki/Expressive_aphasia). |
| Precentral-L,-R | Same to above | Same to above |
| **Brain regions of surface enlargement** |  |  |
| Frontal-Sup-R | Same to above | Same to above |
| Precentral-L,-R | Same to above | Same to above |
| **Brain regions of surface shrink** |  |  |
| Rectus-R | Same to above | Same to above |
| Frontal-Sup-Orb-R | Same to above | Same to above |
| Frontal-Inf-Orb-R |  |  |
| Frontal-Mid-Orb-R |  |  |
| **Temporal lobe** |  |  |
| **Brain regions of volume reduction** |  |  |
| Temporal-Pole-Sup-L,-R | Primary olfactory cortex, smell, emotions. | An impaired sense of [smell](http://en.wikipedia.org/wiki/Olfaction). Disorders of mood as [depression](http://en.wikipedia.org/wiki/Depression_(mood)), [apathy](http://en.wikipedia.org/wiki/Apathy) and [anxiety](http://en.wikipedia.org/wiki/Anxiety). |
| Olfactory-L,-R | Smell. | An impaired sense of [smell](http://en.wikipedia.org/wiki/Olfaction), |
| Fusiform-L,-R | Processing of color information, face and body recognition, word recognition, within-category identification. | Disorders of cognition as parachromatoblepsia or hypochromatopsia, agnosia. Disorders of speech as alexia (Deficits in semantic memory.). |
| Temporal-Inf-L | Recognizing patterns, faces, and objects, visual information processing, The information for color and form, short or long term memory for visual stimuli based on experience. | Prosopagnosia. Cerebral achromatopsia. Disorders of cognition. Deficits in semantic memory. |
| Insula-L | Gustatory cortex, taste. Interoceptive awareness, the control of [blood pressure](http://en.wikipedia.org/wiki/Blood_pressure), sensation of [pain](http://en.wikipedia.org/wiki/Pain), the degree of nonpainful [warmth](http://en.wikipedia.org/wiki/Sense#Temperature) or nonpainful coldness of a skin sensation, stomach or [gastric distension](http://en.wikipedia.org/wiki/Gastric_distension), swallowing, gastric motility. Homeostasis, controls autonomic functions through the regulation of the sympathetic and parasympathetic systems. It has a role in regulating the immune system. The experience of bodily self-awareness, sense of agency, and sense body ownership^,^ the processing of norm violations, emotional processing, empathy, and orgasms. [emotions](http://en.wikipedia.org/wiki/Emotions), including [anger](http://en.wikipedia.org/wiki/Anger), [fear](http://en.wikipedia.org/wiki/Fear), [disgust](http://en.wikipedia.org/wiki/Disgust), [happiness](http://en.wikipedia.org/wiki/Happiness), and [sadness](http://en.wikipedia.org/wiki/Sadness). | Impaired sense of taste, [orthostatic hypotension](http://en.wikipedia.org/wiki/Orthostatic_hypotension), sensation of pain and [paresthesia](http://en.wikipedia.org/wiki/Paresthesia), [Constipation](http://en.wikipedia.org/wiki/Constipation) and [gastric dysmotility](http://en.wikipedia.org/wiki/Gastrointestinal_dysmotility), [oily skin](http://en.wikipedia.org/wiki/Human_skin#Oily_skin) and [excessive sweating](http://en.wikipedia.org/wiki/Hyperhidrosis), [urinary incontinence](http://en.wikipedia.org/wiki/Urinary_incontinence) and [altered sexual function](http://en.wikipedia.org/wiki/Sexual_dysfunction). [Depression](http://en.wikipedia.org/wiki/Depression_(mood)), [apathy](http://en.wikipedia.org/wiki/Apathy) and [anxiety](http://en.wikipedia.org/wiki/Anxiety). |
| **Brain regions of thickness thinning** |  |  |
| Heschl-L,-R | Same to above | Same to above |
| Temporal-Sup-L,-R | Involved in the perception of emotions in facial stimuli, auditory processing, as well as in the function of language in individuals who may have an impaired vocabulary, or are developing a sense of language. involved in social cognition processes, linked to the ability of processing information the many changeable characteristics of a face. | Prosopagnosia. Disorders of auditory sense, speech as anandia, logamnesia, cognition. |
| Temporal-Inf-L |  |  |
| Temporal-Mid-L,-R |  |  |
| Fusiform-L, | Same to above | Same to above |
| **Brain regions of surface enlargement** |  |  |
| Heschl-R | Primary auditory cortex, secondary auditory cortex. Hearing. | Disorders of auditory sense. |
| Temporal-Sup-R |  |  |
| Temporal-Mid-R | Same to above | Same to above |
| **Brain regions of surface shrink** |  |  |
| Temporal-Pole-Sup-R | Visual memories, involved in high-level auditory processing, primary [auditory](http://en.wikipedia.org/wiki/Hearing_(sense)) perception, process the information into meaningful units such as speech and words. Interpret the meaning of visual stimuli and establish object recognition. Language recognition of extend to comprehension, naming, and [verbal memory](http://en.wikipedia.org/wiki/Verbal_memory). [Encoding](http://en.wikipedia.org/wiki/Encoding_(memory)) [declarative](http://en.wikipedia.org/wiki/Declarative_memory) [long term memory](http://en.wikipedia.org/wiki/Long-term_memory). | Unilateral temporal lesion: Contralateral homonymous upper [quadrantanopia](http://en.wikipedia.org/wiki/Quadrantanopia) (sector [anopsia](http://en.wikipedia.org/wiki/Anopsia)), Complex hallucinations (smell, sound, vision, memory); Dominant hemisphere. [Receptive aphasia](http://en.wikipedia.org/wiki/Receptive_aphasia) ([Wernicke's aphasia](http://en.wikipedia.org/wiki/Wernicke%27s_aphasia), [anomic aphasia](http://en.wikipedia.org/wiki/Anomic_aphasia), [dyslexia](http://en.wikipedia.org/wiki/Dyslexia), impaired verbal memory, word agnosia, [word deafness](http://en.wikipedia.org/wiki/Word_deafness)); Non-dominant hemisphere (Impaired non-verbal memory, Impaired musical skills, [prosopagnosia](http://en.wikipedia.org/wiki/Prosopagnosia)) Bitemporal lesions (Additional features): [Deafness](http://en.wikipedia.org/wiki/Deafness), [apathy](http://en.wikipedia.org/wiki/Apathy) (affective indifference), impaired learning and memory. |
| Temporal-Pole-Mid-R |  |  |
| Temporal-Sup-R | Same to above | Same to above |
| Insula-R | Same to above | Same to above |
| Temporal-Inf-R | Perception, vision, reading, speech. | Sensation of pain and [paresthesia](http://en.wikipedia.org/wiki/Paresthesia) (skin tingling and numbness). [Blurred](http://en.wikipedia.org/wiki/Blurred_vision) or [double vision](http://en.wikipedia.org/wiki/Double_vision), visual hallucination. Disorders of speech such as alexia, aphasia. |
| Temporal-Mid-R |  |  |
| **Parietal lobe** |  |  |
| **Brain regions of volume reduction** |  |  |
| Precuneus-L,-R | [Self-consciousness](http://en.wikipedia.org/wiki/Self-consciousness), [episodic memories](http://en.wikipedia.org/wiki/Episodic_memories), [source memory](http://en.wikipedia.org/wiki/Source_amnesia), Visuospatial imagery. | Sensation of pain and [paresthesia](http://en.wikipedia.org/wiki/Paresthesia), Memory is affected, specifically in [recalling](http://en.wikipedia.org/wiki/Recall_(memory)) learned information. Visuospatial difficulties, for example when the individual is asked to perform tests of facial recognition and perception of the orientation of drawn lines. |
| Postcentral-L | Primary somatosensory cortex. Touch sense. Body awareness. | Disorder of somatosensory. leading to [impaired balance](http://en.wikipedia.org/wiki/Balance_disorder) and frequent falls. |
| **Brain regions of thickness thinning** |  |  |
| SupraMarginal-L,-R | [Somatosensory association cortex](http://en.wikipedia.org/wiki/Somatosensory_system), perception of space and limbs location, identifying postures and gestures of other people, controlling our empathy towards other people, phonological word choices. | Disorder of empathy, inhibiting our ability to be empathetic, [impulse control](http://en.wikipedia.org/wiki/Impulse_control_disorders) behaviors such as [medication overuse and craving](http://en.wikipedia.org/wiki/Dopamine_dysregulation_syndrome), [binge eating](http://en.wikipedia.org/wiki/Binge_eating), [hypersexuality](http://en.wikipedia.org/wiki/Hypersexuality), or [pathological gambling](http://en.wikipedia.org/wiki/Pathological_gambling). Speech dysfunction. |
| Postcentral-R | Same to above | Same to above |
| Fusiform-L,-R | Same to above | Same to above |
| Precuneus-L,-R | Same to above | Same to above |
| Parietal-Inf-R | Perception, vision, reading, speech. | Destruction to the inferior parietal lobule of the dominant hemisphere results in [Gerstmann's syndrome](http://en.wikipedia.org/wiki/Gerstmann%27s_syndrome): right-to-left confusion, finger agnosia, dysgraphia and dyslexia, dyscalculia, contralateral hemianopia, or lower quadrantanopia. Destruction to the inferior parietal lobule of the non-dominant hemisphere results in topographic memory loss, anosognosia, construction apraxia, dressing apraxia, contralateral sensory neglect, contralateral hemianopia, or lower quadrantanopia. |
| Angular-R |  |  |
| **Brain regions of surface enlargement** |  |  |
| Precuneus-R | Same to above | Same to above |
| Angular-R | Same to above | Same to above |
| Postcentral-L,-R | Same to above | Same to above |
| Parietal-Inf-L | Perception, vision, reading, speech. | Sensation of pain and [paresthesia](http://en.wikipedia.org/wiki/Paresthesia). [Blurred](http://en.wikipedia.org/wiki/Blurred_vision) or [double vision](http://en.wikipedia.org/wiki/Double_vision), visual hallucination. Disorders of speech as alexia, aphasia, cognition. |
| SupraMarginal-L |  |  |
| **Brain regions of surface shrink** |  |  |
| SupraMarginal-R | Same to above | Same to above |
| Postcentral-R | Same to above | Same to above |
| **Occipital lobe** |  |  |
| **Brain regions of volume reduction** |  |  |
| Occipital-Mid-R | [Visual cortex](http://en.wikipedia.org/wiki/Visual_cortex). [Cuneus](http://en.wikipedia.org/wiki/Cuneus), [lingual,](http://en.wikipedia.org/wiki/Lingual_gyrus) [calcarine gyrus](http://en.wikipedia.org/wiki/Calcarine_sulcus) | See the comment of [Cuneus](http://en.wikipedia.org/wiki/Cuneus), [lingual,](http://en.wikipedia.org/wiki/Lingual_gyrus) [calcarine gyrus.](http://en.wikipedia.org/wiki/Calcarine_sulcus) |
| Occipital-Inf-R |  |  |
| Lingual-L,-R | Vision and dreaming, the identification and recognition of words. | [Blurred](http://en.wikipedia.org/wiki/Blurred_vision) or [double vision](http://en.wikipedia.org/wiki/Double_vision), visual hallucination. Nightmare, Disorders of speech, cognition, such as alexia. |
| Calcarine-L,-R | Primary visual cortex. Vision. | [Blurred](http://en.wikipedia.org/wiki/Blurred_vision) or [double vision](http://en.wikipedia.org/wiki/Double_vision), visual hallucination. |
| **Brain regions of thickness thinning** |  |  |
| Lingual-L,-R | Same to above | Same to above |
| Occipital-Sup-R | [Occipital pole of cerebrum](http://en.wikipedia.org/wiki/Occipital_pole_of_cerebrum), [lateral occipital gyrus](http://en.wikipedia.org/wiki/Lateral_occipital_sulcus), [lunate sulcus](http://en.wikipedia.org/wiki/Lunate_sulcus), [transverse occipital sulcus](http://en.wikipedia.org/wiki/Transverse_occipital_sulcus). | [Homonymous hemianopsia](http://en.wikipedia.org/wiki/Homonymous_hemianopsia) [vision loss](http://en.wikipedia.org/wiki/Vision_loss), [color agnosia](http://en.wikipedia.org/wiki/Color_agnosia), [movement agnosia](http://en.wikipedia.org/wiki/Akinetopsia), and [agraphia](http://en.wikipedia.org/wiki/Agraphia). visual [hallucinations](http://en.wikipedia.org/wiki/Hallucination). |
| Occipital-Inf-R | Same to above | Same to above |
| Occipital-Mid-R |  |  |
| Cuneus-L,-R | Basic visual processing. | [Blurred](http://en.wikipedia.org/wiki/Blurred_vision) or [double vision](http://en.wikipedia.org/wiki/Double_vision), visual hallucination. |
| Calcarine-L,-R | Same to above | Same to above |
| **Brain regions of surface enlargement** |  |  |
| Lingual-R | Same to above | Same to above |
| Occipital-Mid-R | Same to above | Same to above |
| Cuneus-R | Same to above | Same to above |
| Calcarine-R | Same to above | Same to above |
| **Brain regions of surface shrink** |  |  |
| No | No | No |
| **Limbic lobe** |  |  |
| **Brain regions of volume reduction** |  |  |
| Hippocampus-L,-R | Memory, spatial navigation and control of attention. | Memory is affected, specifically in [recalling](http://en.wikipedia.org/wiki/Recall_(memory)) learned information. Visuospatial difficulties, for example when the individual is asked to perform tests of facial recognition and perception of the orientation of drawn lines. Fluctuations in [attention](http://en.wikipedia.org/wiki/Attention) |
| ParaHippocampal-L,-R | Primary olfactory cortex, smell. Emotions. | An impaired sense of [smell](http://en.wikipedia.org/wiki/Olfaction), disorders of mood. |
| Amygdala-L,-R | Emotional learning. Memory modulation. | Disorders of [long-term memory](http://en.wikipedia.org/wiki/Long-term_memory), sexual orientation, social interaction, aggression, fear, anxiety. |
| Cingulum-Ant-L,-R | Involves with emotion formation and processing, learning, and memory. It also plays a role in [executive function](http://en.wikipedia.org/wiki/Executive_function) and respiratory control. | Disorders such as [depression](http://en.wikipedia.org/wiki/Major_depressive_disorder) and [schizophrenia](http://en.wikipedia.org/wiki/Schizophrenia). Disorders of learning, memory, respiratory control. |
| Cingulum-Post-L,-R |  |  |
| **Brain regions of thickness thinning** |  |  |
| ParaHippocampal-L,-R | Same to above | Same to above |
| **Brain regions of surface enlargement** |  |  |
| Cingulum-Post, -Mid, -Ant-R | Same to above | Same to above |
| **Brain regions of surface shrink** |  |  |
| No | No | No |
| **Cerebellum** |  |  |
| **Brain regions of volume reduction** |  |  |
| Cerebellum-Crus1,2-L,-R | Regulates movements of eyes and limbs, helps us to maintain posture and balance, stereotyped movements. | Deficient [ocular pursuit](http://en.wikipedia.org/wiki/Smooth_pursuit) and [saccadic movements](http://en.wikipedia.org/wiki/Saccade), difficulties in directing gaze upward, and [blurred](http://en.wikipedia.org/wiki/Blurred_vision) or [double vision](http://en.wikipedia.org/wiki/Double_vision). [Postural instability](http://en.wikipedia.org/wiki/Postural_instability), [impaired balance](http://en.wikipedia.org/wiki/Balance_disorder) and frequent falls, [Hypokinesia](http://en.wikipedia.org/wiki/Hypokinesia) as writing ([Small handwritin](http://en.wikipedia.org/wiki/Micrographia_(handwriting))g), sewing or getting dressed. |
| Cerebellum-4,-5,-6,-7b,-8,-9-L,-R |  |  |
| Vermis-4,-5,-6,-7,-8,-9,-10 |  |  |
| **Brain regions of thickness thinning** |  |  |
| No | No | No |
| **Brain regions of surface enlargement** |  |  |
| No | No | No |
| **Brain regions of surface shrink** |  |  |
| No | No | No |
| **Other brain regions** |  |  |
| **Brain regions of volume reduction** |  |  |
| Caudate-L,-R | Spatial mnemonic processing integrates spatial information with motor behavior formulation, directed movements, involves in coding a motor response, in the recruitment of the motor system to support working memory performance by the mediation of sensory-motor transformations, contributes importantly to body and limbs posture and the speed and accuracy of directed movements, executive functioning, working memory, sleep correlated with a decrease in the duration of deep [slow wave sleep](http://en.wikipedia.org/wiki/Slow_wave_sleep), total volume of deep slow wave sleep, emotion implicated in responses to visual beauty, threshold control prevent explosive activation. | [Tremor](http://en.wikipedia.org/wiki/Tremor), [postural instability](http://en.wikipedia.org/wiki/Postural_instability), daytime [drowsiness](http://en.wikipedia.org/wiki/Somnolence), disturbances in [REM](http://en.wikipedia.org/wiki/Rapid_eye_movement_sleep) sleep, or [insomnia](http://en.wikipedia.org/wiki/Insomnia). Disorder of moods as [depression](http://en.wikipedia.org/wiki/Depression_(mood)), [apathy](http://en.wikipedia.org/wiki/Apathy) and [anxiety](http://en.wikipedia.org/wiki/Anxiety). |
| Thalamus-L,-R | Every sensory system (With the exception of the [olfactory system](http://en.wikipedia.org/wiki/Olfactory_system)), such as the [auditory](http://en.wikipedia.org/wiki/Auditory_system), [somatic](http://en.wikipedia.org/wiki/Somatic_sensory_system), [visceral](http://en.wikipedia.org/wiki/Visceral), [gustatory](http://en.wikipedia.org/wiki/Gustatory) and [visual systems](http://en.wikipedia.org/wiki/Visual_system). Episodic memory. Regulating states of [sleep and wakefulness](http://en.wikipedia.org/wiki/Neuroscience_of_sleep), [consciousness](http://en.wikipedia.org/wiki/Consciousness). | The complex symptomes of sensory system including the [auditory](http://en.wikipedia.org/wiki/Auditory_system), [somatic](http://en.wikipedia.org/wiki/Somatic_sensory_system), [visceral](http://en.wikipedia.org/wiki/Visceral), [gustatory](http://en.wikipedia.org/wiki/Gustatory) and [visual systems](http://en.wikipedia.org/wiki/Visual_system). The different level of dementia. Several sleep disorders. |
| **Brain regions of thickness thinning** |  |  |
| No | No | No |
| **Brain regions of surface enlargement** |  |  |
| No | No | No |
| **Brain regions of surface shrink** |  |  |
| No | No | No |
